# Sediment Quality of the SW Coastal Laizhou Bay, Bohai Sea, China: A Comprehensive Assessment Based on the Analysis of Heavy Metals

**DOI:** 10.1371/journal.pone.0122190

**Published:** 2015-03-27

**Authors:** Xuelu Gao, Wen Zhuang, Chen-Tung Arthur Chen, Yong Zhang

**Affiliations:** 1 Key Laboratory of Coastal Environmental Processes and Ecological Remediation, Yantai Institute of Coastal Zone Research, Chinese Academy of Sciences, Yantai, Shandong, China; 2 Department of Oceanography, National Sun Yat-Sen University, Kaohsiung, Taiwan; 3 College of City and Architecture Engineering, Zaozhuang University, Zaozhuang, Shandong, China; University of California, Merced, UNITED STATES

## Abstract

Historically, the Bohai Sea is one of the most important fishing grounds in China. Yet, surrounded by one of the biggest economic rims of China, its ecological functions have been declining rapidly in recent two decades under the heavy anthropogenic impacts. The Laizhou Bay is the smallest one of the three main bays in the Bohai Sea. Owing to the rich brine deposits, chemical industries using brine as raw materials are booming in the southern coast of the Laizhou Bay, the scale of which ranks as the largest one in China. In order to monitor and assess the environmental quality, surface sediments were collected from the coastal waters of southwestern Laizhou Bay and the rivers it connects with during summer and autumn in 2012, and analyzed for heavy metals. Several widely adopted methods were used in the overall assessment of heavy metal pollution status and potential ecological risks in these sediments, and the data were analyzed to infer the main sources of the pollutants. The results showed that the remarkably high concentrations of heavy metals were almost all recorded in a small number of riverine sites. Cr, Cu, Ni and Zn were the main environmental threat according to the sediment quality guidelines. The marine area was generally in good condition with no or low risk from the studied metals and adverse effects on biota could hardly occur. Natural sources dominated the concentrations and distributions of Cu, Ni, Pb and Zn in the marine area. Our results indicated that heavy metal pollution was not a main cause of the ecological degradation of the Laizhou Bay at present.

## Introduction

**Fig 1:**
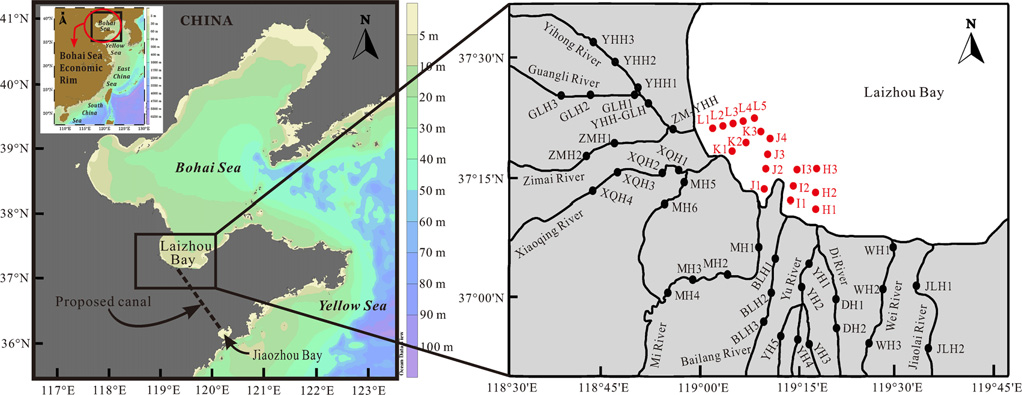
Location of sampling sites in the southwestern coastal Laizhou Bay. The riverine sampling sites were shown in black and the marine sampling sites were shown in red.

Although there is quite a controversy about the main causes and what kind of management measures should be taken, one fact that cannot be denied is that the eco-environmental condition of the Bohai Sea, which was once one of the most important fishing grounds in China, has degraded significantly under the influence of massive human activities [1]. Many suggestions have been proposed to save the ecosystem of the Bohai Sea, among which the most ambitious one was probably the one of accelerating the water circulation rate of the Bohai Sea by connecting it with the Jiaozhou Bay in the west coast of the southern Yellow Sea through an interbasin canal starting from the southern coastline of the Laizhou Bay (Fig. 1) [2]. Billions of dollars have been spent in remediating and conserving the ecological functions of the Bohai Sea, conducting the environmental monitoring and surveillance, and building the warning and emergency systems [3]. Surrounded by one of the biggest economic rims of China, evidence shows that metal contamination in the Bohai Sea is closely associated with the fast economic growth of its surrounding areas during the past decades [3].

Excessive input of heavy metals is a serious environmental problem in many coastal ecosystems around the world, and this has received extensive attention because many heavy metals are toxic, non-biodegradable in environment and easy to accumulate in organisms [4–7]. The coastal marine sediments act as filters trapping both natural and anthropogenic materials including heavy metals transferred from the continents to the open seas; however, when environmental conditions change, sediments may transform from the main sink of heavy metals to the sources of them for the overlying waters [8, 9]. Therefore, the contents of heavy metals in sediments are widely monitored to provide basic information for the environmental risk assessment [3, 10].

The Laizhou Bay is one of the three major bays in the Bohai Sea, making up ~10% of its total areas, with a coastline length of ~320 km and a surface area of ~7000 km^2^. It used to be one of the most important spawning and breeding grounds for many marine organisms in China. There are over a dozen small rivers running into the Laizhou Bay mainly from its southwestern coast such as the Guangli River, the Xiaoqing River, the Bailang River and so on. The Laizhou Bay is a continental climate area of the warm temperate semi-humid monsoon type. The annual average temperature is 11.9~12.6°C. The highest temperature appears in July, with the monthly average values of 25.9~26.4°C; the lowest appears in January, with the monthly average values of -2.8~-3.8°C. The average annual rainfall is 670~800 mm. Rainfall focuses between late June and early September, accounting for about 72%~76% of the overall rainfall. The Laizhou Bay is an irregular mixed semidiurnal tide area, and the flood and ebb last for 6.4 h and 6.0 h, respectively. The average tidal range of the Laizhou Bay is 0.9 m. Velocities of flood and falling tides are 29~37 cm s^-1^ and 29~39 cm s^-1^, respectively.

In recent years, the Laizhou Bay is under unprecedented threats because of the rapid development of urbanization and industrialization, overfishing and the unreasonable use of the coastline. Brine resources are rich in the southwestern coastal Laizhou Bay, due to which over 400 chemical enterprises are located along the southwestern coastline of the Laizhou Bay, forming one of the biggest manufacturing bases for chlor-alkali products in the world. The development of economy brings serious ecological threats to the Laizhou Bay, and its fishery resources have gradually declined. Coastal ecosystems that are close to industrialized communities, a situation that the Laizhou Bay is in, are likely to receive more heavy metal loadings from river discharges, inlets and estuaries filled with run-off from adjacent lands [11].

The fact that sediments integrate the external environmental impacts makes them an essential source for information acquisition in major marine monitoring programs. To sum up, the purposes of this study are i) to investigate the spatial and seasonal distributions of the concentrations and fractionations of six heavy metals, i.e. Cd, Cr, Cu, Ni, Pb and Zn, that relate to the environmental quality assessment in the surface sediments of the southwestern marine area of the Laizhou Bay and the major rivers it connects with, ii) to estimate the anthropogenic influence, and iii) to assess the degree of contamination and the ecological risks of these heavy metals to the environment. The southwestern coast of the Laizhou Bay was chosen because it is the area of the Laizhou Bay facing the most serious threat from anthropogenic activities [12–14].

## Materials and Methods

### Ethics statement

This study did not involve endangered or protected species and no specific permissions were required for these locations/activities in this study. The specific locations of the present study were shown in Fig. 1.

### Sampling

The field sampling work of this research was carried out twice in 2012: early summer (May–June, hereafter referred to as summer for short), which was before the peak period of the rainy season, and middle autumn (September–October, hereafter referred to as autumn for short), which was after the peak period of the rainy season. The sampling sites were arranged along the major rivers of this area extending from the land to the sea and formed five transects, covering about 15–20 km from the high tide mark to the land and about 10 km from the high tide mark to the sea (Fig. 1). A total of 53 surface sediment samples (top ~5 cm) were collected in summer and 51 were collected in autumn (Fig. 1) (samples from sites MH3 and YHH2 were not collected in autumn because these two sites were unreachable), among which 18 marine samples were from the same sites in both sampling periods. The riverine surface sediments were collected using a plastic spatula from the shallow water near the riverside, and the marine surface sediments were collected with a stainless steel grab sampler. The samples used in this study were placed in acid cleaned polyethylene bags, and stored in a cooler box with ice bags immediately after collection. When taken back to the lab, the samples were stored at ~4°C in the dark until analyses were made.

### Analytical methods

The information about the fractionations of metals in the surface sediments was obtained by a sequential extraction procedure reported by Rauret et al. [15]. The four operationally defined geochemical fractions separated by this scheme are acid soluble (F1), reducible (F2), oxidizable (F3) and residual (F4) fractions. The detailed sequential extraction protocol used in this study has been described elsewhere [16]. It has been reported that the process of drying could alter the solid phase distribution of trace elements [17, 18]; besides, the elemental concentrations in sediments are highly dependent on the grain size [19, 20], so triturating treatment could potentially alter the extractability of elements [21]. Therefore, wet and unground sediments were used for the sequential extraction procedure in this study to reflect as much as possible the actual situation in natural environment.

The total concentration of each studied metal in all samples was obtained by digesting an aliquot of freeze-dried, homogenized and ground sediment with the mixture of concentrated HF, HNO_3_ and HClO_4_ (5:2:1) [16]. The concentrations of metals in residual fractions were calculated by subtracting the metal concentrations of the first three steps of the sequential extraction from the total digestion measurements. Fifteen randomly selected residues from the 3rd step of the extraction procedure were digested with the same method as the total digestion measurements, and the sums of the measured values of the four geochemical fractions accounted for 85–110% of the corresponding values from the total digestion measurements. Inductively coupled plasma mass spectrometry (PerkinElmer Elan DRC II) was applied in this work for the determination of Cd, Cr, Cu, Ni, Pb and Zn. In addition, the concentrations of Al and Fe were analyzed by inductively coupled plasma optical emission spectrometer (PerkinElmer Optima 7000 DV). The results of Al were used to normalize the data of the studied heavy metals to separate the metals of natural variability from the fraction that is associated with sediments due to human activities by calculating their enrichment factors (EFs). The quality control was given below.

Analytical methods and results of the total organic carbon (TOC), grain size and moisture content (MC) of the samples have been described in previous publications [14, 22–24]. The substance concentrations of sediments were expressed on the dry weight basis based on the results of MC.

### Quality control

The analytical data quality was guaranteed through the implementation of laboratory quality assurance and quality control methods, including the use of standard operating procedures, calibration with standards, analysis of reagent blanks, and analysis of replicates. The quality of the analytical procedures was tested by recovery measurements on the Chinese national geostandard samples (GBW-07333 and GBW-07314). The results were consistent with the reference values, the differences were all within ±10%, and the precision, expressed as the relative standard deviation (RSD), ranged from 5% to 10%. The precision of the analysis of standard solution (RSD) was better than 5%. All analyses were carried out in duplicate, and the results were expressed as the mean. All reagents were analytical or guaranteed grade. All the labwares (bottles, tubes, etc.) were pre-cleaned by soaking in 10% HNO_3_ (w/w) for at least 2 days, followed by soaking and rinsing with de-ionized water.

### Assessment of sediment contamination and ecological risk

In this study, two sediment quality guidelines (SQGs) [25, 26] and three typical environmental indices [27–31] were used to assess the degree of heavy metal contamination and ecological risks in the surface sediments of the Laizhou Bay. The specific details of the SQGs and the indices are shown in Tables 1 and 2, respectively. It is an ideal way to compare the levels of metals with their corresponding pre-industrial reference levels. However, no such background data are available for each individual estuary around the Laizhou Bay. Therefore, the average upper continental crust (UCC) values [32] were chosen as the reference background values for the studied metals in the calculation of related indices in this study. The choice of UCC is justified by the fact that the surface sediments in the southwestern Laizhou Bay are from the Yellow River which discharges huge amounts of loess into the sea each year and the local rivers to the southwest of the Laizhou Bay [33], and the southwestern coast of the Laizhou Bay is a part of a littoral plain with thick sediments [34].

**Table 1:** The metal guideline values of two sediment quality guidelines (SQGs) used to distinguish marine sediment quality (μg g^-1^).

Sediment quality guidelines	Cd	Cr	Cu	Ni	Pb	Zn	Reference
Class I upper limit	0.5	80	35	nd^a^	60	150	[25]
Class II upper limit	1.5	150	100	nd	130	350	[25]
Class III upper limit	5	270	200	nd	250	600	[25]
TEL guideline	0.68	52.3	18.7	15.9	30.2	124	[26]
PEL guideline	4.2	160	108	42.8	112	271	[26]

^a^ nd: not defined.

**Table 2:** The summary of three normally used indices for the assessment of heavy metal contamination and environmental risk in sediment.

Index	Description	Category		References
Geoaccumulation index (*I*_geo_)	$I_{geo}=\log_{2}\left(\frac{C_{n}}{1.5B_{n}}\right)$. *C*_n_ is the measured concentration of metal n; *B*_n_ is the geochemical background concentration of metal n. Correction index 1.5 is usually used to take into consideration possible differences in the background values due to lithological variation.	*I*_geo_ ≤ 0	Practically uncontaminated	[27, 28]
		0 < *I*_geo_ ≤ 1	Uncontaminated to moderately contaminated	
		1 < *I*_geo_ ≤ 2	Moderately contaminated	
		2 < *I*_geo_ ≤ 3	Moderately to heavily contaminated	
		3 < *I*_geo_ ≤ 4	Heavily contaminated	
		4 < *I*_geo_ ≤ 5	Heavily to extremely contaminated	
		5 < *I*_geo_	Extremely contaminated	
Enrichment factor (EF)	$EF=\frac{{\left(C_{\text{X}}/C_{\text{A1}}\right)}_{\text{Sample}}}{{\left(C_{\text{X}}/C_{\text{A1}}\right)}_{\text{UCC}}}$. *C*_x_ and *C*_Al_ denote the concentrations of element x and Al in the samples and in UCC, respectively. In this study, Al was used as the reference element for geochemical normalization because it represents the quantity of aluminosilicates which is generally the predominant carrier phase for metals in coastal sediments and its natural concentration tends to be uniform.	EF ≤ 1	No enrichment	[29, 30]
		1 < EF ≤ 3	Minor enrichment	
		3 < EF ≤ 5	Moderate enrichment	
		5 < EF ≤ 10	Moderately severe enrichment	
		10 < EF ≤ 25	Severe enrichment	
		25 < EF ≤ 50	Very severe enrichment	
		50 < EF	Extremely severe enrichment	
Risk assessment code (RAC)	*RAC* = *Exc*% + *Carb*%. *Exc*% and *Carb*% are the percentages of metals in exchangeable and carbonate fractions, i.e. acid soluble fraction in the present study.	RAC ≤ 1%	No risk	[31]
		1% < RAC ≤ 10%	Low risk	
		10% < RAC ≤ 30%	Medium risk	
		30% < RAC ≤ 50%	High risk	
		50% < RAC	Very high risk	

The National Standard of China GB18668-2002 [25] has defined three grades of marine sediments, in which the concentrations of five heavy metals that were measured in this study, i.e. Cd, Cr, Cu, Pb and Zn, are regarded as parameters used to classify the marine sediment quality. Without considering other factors, if the concentration of a heavy metal in sediments of a marine area meets the first class quality of GB18668-2002 (Class I), the area is suitable for mariculture, nature reserve, endangered species reserve and leisure activities; if it meets the second class quality of GB18668-2002 (Class II), the area is suitable for industry and tourism site; and if it meets the third class quality of GB18668-2002 (Class III), the area can just be used for harbor (Table 1).

Threshold effects level (TEL) and probable effects level (PEL) are guidelines widely used to assess bio-toxic risks of sediments [26]. TEL is the concentration of an element or a compound in sediment below which adverse biological effects rarely occur; PEL is the concentration of an element or a compound in sediment above which adverse biological effects frequently occur (Table 1).

In view of the fact that contaminants always occur in sediments as complex mixtures, the mean PEL quotient method has been proposed and adopted to determine the possible biological effect of combined toxicant groups by calculating the mean quotients for a range of contaminants using the following formula [35, 36]:

$$Mean\;PEL\;quotient=\frac{\sum_{x=1}^{n}\frac{C_{x}}{PEL_{x}}}{n}$$

where *C*_*x*_ is the sediment concentration of component *x*, *PEL*_*x*_ is the PEL for component *x* and *n* is the sum of components. For marine sediments, the mean PEL quotients of <0.1, 0.11–1.5, 1.51–2.3 and >2.3 indicate that the combined effects of contaminants have an 8%, 21%, 49% and 73% probability of being biologically toxic, respectively [36].

### Statistical Analysis

Statistical methods were applied to process the analytical data in terms of the distribution and relationships among the studied parameters. Pearson’s correlation coefficient analysis was performed to identify the relationship among heavy metals in sediments and their possible sources. The principal component analysis (PCA) was performed to extract significant principal components (PCs) and further reduce the contribution of variables with minor significance. The PCA was performed with varimax rotation with Kaiser Normalization to generate varifactors (VFs) in this study. The commercial statistics software package SPSS version 19.0 for Windows was used for statistical analyses mentioned above. Scatter graphs were drawn using Origin version 8 to determine whether there were significant differences in sources between each studied metal in different seasons. The agglomerative hierarchical clustering (AHC) analysis was conducted on the normalized data set using Ward’s method with Euclidean distances as a measure of dissimilarity to assess the interrelationships among the sampling sites. The XLSTAT software (version 2013) was used in the AHC analysis.

## Results and Discussion

### Metals in total concentrations

**Fig 2:**
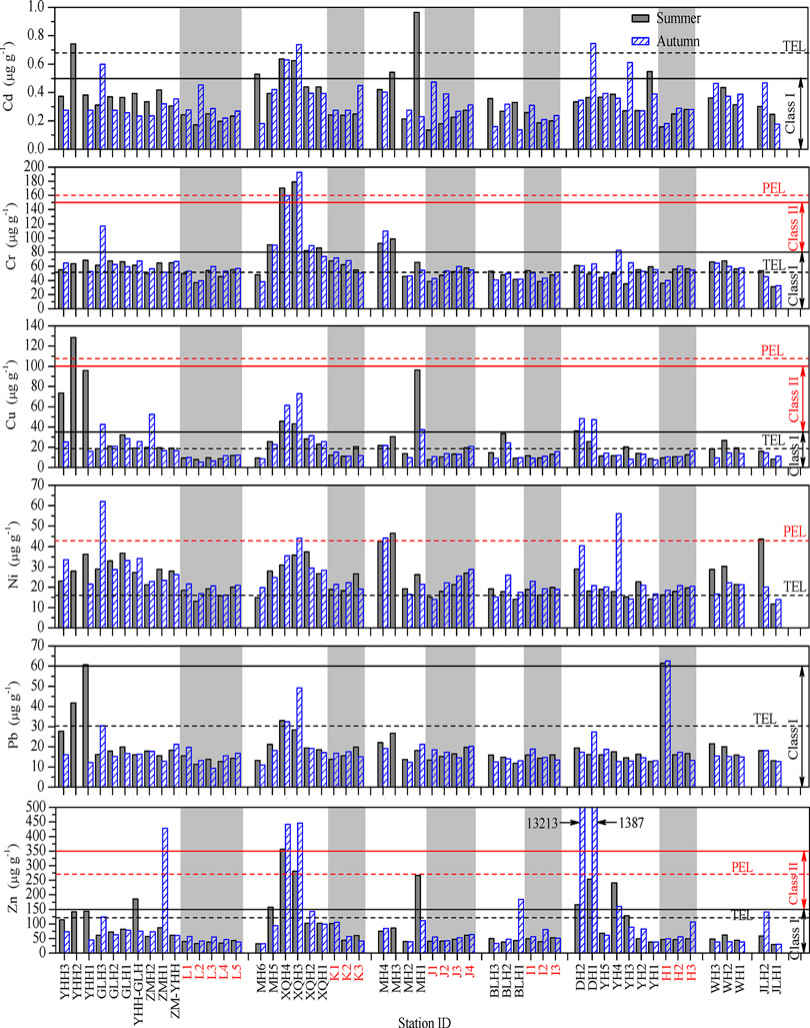
The spatial variations of the total concentrations of each studied metal in the surface sediments of the southwestern coastal Laizhou Bay. The horizontal dash lines represent their corresponding TEL or PEL concentrations; the horizontal solid lines represent their corresponding higher boundary values of Class I or Class II sediment categories of China. The data of the samples from the marine sampling sites were indicated with light grey background and red labels. Related data can be found in S1 Table.

The spatial distribution of heavy metals is shown in Fig. 2 and the related information is summarized in Table 3. Except that the mean concentrations of Pb in the marine sediments (18.6 μg g^-1^) were slightly higher than that in the riverine sediments (17.9 μg g^-1^) in autumn, the mean concentrations of all the other studied metals in the marine sediments were much lower than that in the riverine sediments in both seasons. The highest concentrations of Pb in both seasons were found in the surface sediment of site H1 which was located in the marine area (summer, 61.5 μg g^-1^; autumn, 62.6 μg g^-1^), and the concentrations of Pb in the surface sediments of all the rest of marine sites and most of the riverine sites were much lower, and almost all lower than its corresponding value in the UCC, i.e. 20 μg g^-1^ (Fig. 2; Table 3). Site H1 was close to the coast, the high Pb concentration might be caused by industrial effluent discharge from hidden drain outlets.

**Table 3:** Heavy metal concentrations in the surface sediments of the southwestern coastal Laizhou Bay. The related values reported for the surface sediments of other marine areas and the average upper continental crust (UCC) values are shown for comparison. Concentration unit is μg g^-1^ for all elements.

Location	Sampling date	Sample size		Cd	Cr	Cu	Ni	Pb	Zn	References
Rivers in the SW coastal Laizhou Bay (Bohai Sea), China	May-Jun., 2012	35	Range	0.21–0.96	31.0–179	8.3–129	11.6–46.6	11.8–60.7	29.2–357	This study
			Mean	0.41	67.2	30.2	26.4	20.3	109	
	Sep.-Oct., 2012	33	Range	0.14–0.75	32.4–192	7.3–72.9	14.0–62.0	11.1–49.2	30.2–13214	
			Mean	0.37	68.9	23.9	27.0	17.9	548 (113^a^)	
Coastal waters in the SW Laizhou Bay (Bohai Sea), China	May-Jun., 2012	18	Range	0.13–0.28	36.2–67.8	7.6–20.6	13.1–27.0	11.4–61.5	33.5–101	This study
			Mean	0.22	50.6	11.5	18.9	17.9	48.3	
	Sep.-Oct., 2012	18	Range	0.18–0.47	39.7–71.7	5.1–20.7	14.0–28.8	9.4–62.6	37.3–107	
			Mean	0.30	53.5	11.9	20.6	18.6	58.7	
Laizhou Bay (Bohai Sea), China	May, 2007	31		0.081	57.1	13.3	19.4	20.2	59.4	[37]
Laizhou Bay (Bohai Sea), China	May, 2008	30		0.11	na^b^	15.0	na	11.7	50.8	[38]
Laizhou Bay (Bohai Sea), China	Oct., 2011	18		0.22	56.7	12.0	25.9	19.4	41.5	[23]
Intertidal Bohai Bay (Bohai Sea), China	May, 2008	15		0.12	68.6	24.0	28.0	25.6	73.0	[39]
Southern Bohai Bay (Bohai Sea), China	Aug., 2008	119		0.14	33.5	22.7	30.5	21.7	71.7	[40]
Coastal Bohai Bay (Bohai Sea), China	May, 2008	42		0.22	101.4	38.5	40.7	34.7	131.1	[10]
Jinzhou Bay (Bohai Sea), China	Oct., 2009	25		26.8	na	74.1	43.5	124.0	689.4	[41]
Liaodong Bay (Bohai Sea), China	2009	128		na	46.4	19.4	22.5	31.8	71.7	[42]
Coastal Shandong Peninsula (Yellow Sea), China	2007	208		na	57.8	20.0	31.2	28.4	74.7	[7]
North Yellow Sea, China	Jun., 2006	278		0.09	48.9	15.9	22.0	24.1	57.3	[43]
South Yellow Sea, China	Oct., 2006	33		0.30	na^b^	16.9	na	17.8	93.7	[5]
Changjiang Estuary (East China Sea), China	Apr. and Aug., 2009	59		0.26	78.9	30.7	31.8	27.3	94.3	[44]
NW East China Sea, China	May, 2009	35		0.30	84.2	33.1	36.1	28.0	102.4	[45]
Shantou Bay (South China Sea), China	Dec., 2010	15		0.67	53.6	48.5	23.0	51.6	153.3	[46]
Daya Bay (South China Sea), China	Jan., 2006	9		0.052	na	20.8	31.2	45.7	113	[16]
Northern South China Sea	Sep., 2005	4		0.110	na	14.1	22.6	18.0	69.9	[47]
Jade Bay (North Sea), Germany	2009–2010	248		0.25	49	7	10	16	43	[48]
İzmit Bay (Marmara Sea), Turkey	Apr. 2002	34		5.10	75.0	66.4	41.2	104.7	961	[49]
The Atlantic and Cantabric coasts, Spain	2001–2007	65		na	na	115	na	91	230	[50]
Gironde Estuary (French Atlantic coast), France	1998–2007	323		0.48	78.4	24.5	31.7	46.8	168	[51]
UCC				0.098	35	25	20	20	71	[32]

^a^ mean value excluding the two extremely high values of the sites DH1 and DH2.

^b^ na: not available.

In general, the relatively higher concentrations of heavy metals were found mainly at several riverine sites in the Yihong, the Guangli, the Xiaoqing, the Mi, the Di and the Yu Rivers that are affected by extensive human activities (Fig. 2). This might be owing to the effects of many and various human activities in the area of this study such as untreated sewage and industrial effluent discharge, the use of pesticides and fertilizers in agriculture, as well as automobile exhaust from heavy traffic and coal burning in power generation plants, all of which can cause the increase of the concentrations of heavy metals in sediments [46, 52, 53].

The Yihong and the Gangli Rivers flow through the urban areas of Dongying City, so heavy metals (especially Cu and Ni) in sediments of these rivers might be attributed to the point sources such as industrial effluent and domestic sewage discharged directly into the rivers [52] and the non-point sources such as traffic activities [53]. In the Xiaoqing River, site XQH3 was at a ferry pier and XQH4 located at the downstream side of a nearby bridge, so heavy metals (especially Cr, Ni and Zn) in the sediments of these sites might be attributed to traffic activities and boat maintenance and repair [52, 53]. The Mi River flows through an industrial zone of this area, so heavy metals (especially Cd, Cu and Zn) in its surface sediments might result from effluent discharge from factories and traffic activities.

By comparing the data obtained in the two sampling periods, the mean concentrations of Cd, Cu and Pb in the riverine sediments decreased slightly from summer to autumn, and during the same time the mean concentrations of Cr and Ni in the riverine sediments increased slightly (Table 3). The mean concentrations of Zn in the riverine sediments increased significantly from summer to autumn (summer, 109.2 μg g^-1^; autumn, 548.4 μg g^-1^) (Table 3). Extremely high concentrations of Zn were found in DH1 and DH2 in autumn, and the corresponding values were up to 1387 μg g^-1^ and 13214 μg g^-1^, respectively (Fig. 2). River bottoms are dynamic places, with both scour and sedimentation occurring. Therefore, certain sediments at any sites in time may capture old sediments if things are recently scoured or capture new sediments if things are deposited recently, or a mix of the two. So changes of heavy metal concentrations in the same sediments could be the results of different hydrodynamic conditions with seasonal changes. However, such a big change in concentrations could not possibly be caused only by natural factors, and it had to be attributed to the influence of factory discharge. Several chemical plants are located in the upstream areas of DH2, and an outfall was found near this site, but it could not be determined to which chemical plant it belonged. It is reported that metals which originated from anthropogenic sources are more loosely bound to sediments than from natural sources, and might be released back to the aqueous phase with the change of environmental physical and chemical conditions [54]. Therefore, the pollution risk of anthropogenic heavy metals to aquatic organisms is higher than natural ones. The sediment influx to the Bay could be from either the mobilization of new sediments or contaminated legacy deposits, or a mix of the two. But there was no data about column sediments or dating, so it could not be determined yet. If the concentrations of Zn in the surface sediments of sites DH1 and DH2 were excluded in the calculations, the mean concentration of Zn in riverine sediments in autumn was only 112.8 μg g^-1^, which was very close to its mean concentration 109.2 μg g^-1^ in summer (Table 3).

So on the whole, there was no significant difference between spatial distribution of the heavy metals in the two sampling periods, and very high concentrations of certain heavy metals in some riverine sediments could be attributed to intensive anthropogenic activities. The mean concentrations of all studied metals in the marine sediments increased slightly from summer to autumn. This was probably because most of the studied rivers are seasonal rivers, and the flows of these rivers increased during the rainy season, so larger amounts of heavy metals were carried from the land to the Laizhou Bay which caused the increase of heavy metal concentrations in the marine sediments in autumn.

**Fig 3:**
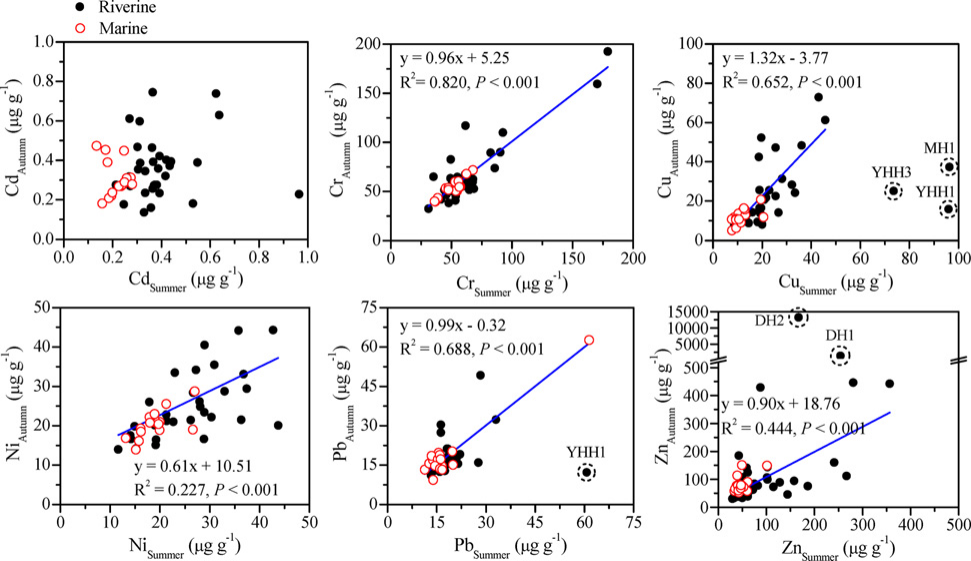
Relationship between the concentrations of each studied metal in the surface sediments of the southwestern coastal Laizhou Bay in summer and autumn. The regression lines were plotted based on the whole data sets (riverine + marine), and the outliers with dashed circles were excluded from the calculations.

Compared with the strong mobility of water, heavy metal contents in sediment are relatively stable. So heavy metal contents of the two sampling periods could largely reflect the situation of the two seasons. To further seek the relationships between the data of the two sampling periods and find out whether there were seasonal differences in sources, scatter graphs were made for each of the studied heavy metals (Fig. 3). It clearly showed that there was no significant correlation between the concentrations of Cd in summer and autumn, which meant that the sources of Cd were much different between summer and autumn. This was because Cd is a typical anthropogenically discharged element and its background value in the natural environment like UCC is at least one order of magnitude lower than the other studied metals, so it was reasonable that its concentration and distribution in an area facing pressure from extensive and intensive human activities like the coastal Laizhou Bay were irregular between the two seasons. There were significant correlations between the concentrations of Cr and Ni in summer and their corresponding concentrations in autumn, indicating that their sources in the studied area were stable and they were mainly from non-point sources and the emission intensity of point sources of them were comparable in summer and autumn. Significant correlations between the concentrations of Cu, Pb and Zn in summer and their corresponding concentrations in autumn could also be observed when very few data from certain riverine sampling sites (e.g. the data of Cu at sites MH1, YHH1 and YHH3) were excluded in the correlation analysis. So the sources of Cu, Pb and Zn were also generally stable and also they were mainly from non-point sources, but the influence of emissions from some point sources was rather considerable considering their high concentrations in the surface sediments of very few riverine sampling sites in summer or autumn (Fig. 3). Taking the studied area as a whole, reflected by the slope and intercept of the linear regression equation in Fig. 3, the difference between the concentrations of Cr in the two sampling seasons for a certain sampling site was small, and this was also true for the concentrations of Pb.

The mean concentrations of Cd and Cr in both the riverine and marine sediments in both seasons were clearly higher with respect to their corresponding values in the UCC; the mean concentration of Cu in the riverine sediments in summer was higher comparing with its corresponding value in the UCC, while its mean values in the riverine sediments in autumn and in the marine sediments in both seasons were lower comparing with its corresponding value in the UCC; the mean concentrations of Ni and Zn in the riverine sediments in both seasons were higher with respect to their corresponding values in the UCC, while their mean values in the marine sediments in both seasons were lower than their corresponding values in the UCC; the mean concentrations of Pb in both riverine and marine sediments in both seasons were very close to its corresponding value in the UCC (Table 3).

Among the three bays of the Bohai Sea, the mean concentrations of heavy metals in surface sediments of the SW Laizhou Bay were the lowest. In addition to the influence of human activities, particle size effect was likely to be one of the main factors for this phenomenon. Surface sediments of the studied marine area were made up of silt-sand or sand-silt [14]. Comparing with the Bohai Bay and the Liaodong Bay whose sediments were made of clay-silt and clay-silt-sand, respectively [10, 42], adsorption ability of surface sediments in the SW Laizhou Bay was weaker. The joint effects of the tidal and residual currents and the anti-clockwise circulation of the Laizhou Bay caused the re-suspension of the fine surface sediments near the estuaries and then drove them to the middle of the Bay, so only the coarse particles with weak adsorption force were left [14]. The mean concentrations of the studied heavy metals in the studied area were far lower with respect to their corresponding values in surface sediments of the Jinzhou Bay in the northern Bohai Sea in China [41], the İzmit Bay in Turkey [49], the Atlantic and Cantabric coasts in Spain [50] and the Gironde Estuary in France [51] which were much heavily polluted coastal zones in the world, while they were higher than their corresponding values in surface sediments of the Jade Bay in Germany [48] where sediment quality was in good condition. Considering only the marine area of this study, the mean heavy metal concentrations were comparable with and in the lower parts of the ranges of their corresponding values in surface sediments of the other marine areas in China such as the Bohai Bay [10, 39, 40], the Liaodong Bay [42], the North and South Yellow Seas [5, 7, 43], the Changjiang Estuary and the NW East China Sea [44, 45], the Daya Bay [16], the Shantou Bay [46], and the northern South China Sea [47], and they were consistent with the results of previous investigations carried out in the offshore areas of the Laizhou Bay [23, 37, 38].

The correlation matrix for the concentrations of the geochemical parameters studied is shown in Table 4. The detailed information about the grain size, TOC and MC in the surface sediments of the studied area has been described elsewhere [14]. All the metals were significantly correlated with each other in both sampling periods, suggesting a major common origin. It has been reported that the deposition of fine grained materials and organic matters physically controls the abundance and distribution of metals in sediments [55]. In summer, all the metals were significantly correlated with TOC; except Cd and Pb, all the other four studied metals were significantly and positively correlated with fine fractions (clay and/or silt) and were significantly and negatively correlated with sand fraction. In summer, the concentration of Pb appeared to be more influenced by the fine grain size composition than by the coarse grain size composition; the concentration of Cd did not appear to be influenced by the grain size composition, perhaps because it is a typical anthropogenically discharged element [10]. In autumn, the grain size controlled the abundance and distribution of all the studied metals except Pb in the surface sediments of the studied area, and meanwhile the concentrations of Cd, Cu and Zn appeared to be influenced by the amount of organic matters. The concentrations of all the metals appeared to be influenced by the moisture content of the sediment in both sampling periods.

**Table 4:** Pearson correlation matrix for the sediment components.

Sampling period		Cd	Cr	Cu	Ni	Pb	Zn	Clay	Slit	Sand	MC	TOC
Summer	Cd	1	0.558^a^	0.677^a^	0.431^a^	0.239	0.637^a^	0.200	0.163	-0.181	0.371^b^	0.438^a^
	Cr		1	0.301^c^	0.599^a^	0.282^c^	0.641^a^	0.363^b^	0.394^b^	-0.407^b^	0.499^a^	0.684^a^
	Cu			1	0.390^b^	0.579^a^	0.511^a^	0.170	0.372^b^	-0.340^c^	0.467^a^	0.413^b^
	Ni				1	0.353^b^	0.312^c^	0.632^a^	0.581^a^	-0.625^a^	0.391^b^	0.465^a^
	Pb					1	0.297^c^	0.137	0.272^c^	-0.252	0.403^b^	0.337^c^
	Zn						1	0.275^c^	0.296^c^	-0.307^c^	0.533^a^	0.566^a^
Autumn	Cd	1	0.633^a^	0.499^a^	0.324^c^	0.347^c^	0.478^a^	0.213	0.287^c^	-0.282^c^	0.435^a^	0.297^c^
	Cr		1	0.724^a^	0.669^a^	0.494^a^	0.689^a^	0.395^b^	0.418^b^	-0.430^b^	0.394^b^	0.062
	Cu			1	0.562^a^	0.491^a^	0.645^a^	0.432^b^	0.581^a^	-0.572^a^	0.666^a^	0.316^c^
	Ni				1	0.283^c^	0.400^b^	0.452^a^	0.514^a^	-0.521^a^	0.311^c^	0.007
	Pb					1	0.380^b^	0.096	0.111	-0.112	0.294^c^	0.093
	Zn						1	0.328^c^	0.322^c^	-0.333^c^	0.497^a^	0.344^c^

^a^ *p* < 0.001.

^b^ 0.001 < *p* < 0.01.

^c^ 0.01 < *p* < 0.05.

### Metal fractionation

The toxicity and mobility of heavy metals in sediments vary greatly among different geochemical forms, and heavy metal fractionation offers a more realistic evaluation of their actual environmental impact [10, 52]. Fractionation analysis can provide information on the strength and ways of metals associating with the sediment matrix and thus can be used to assess the possible metal impact on biota in aquatic ecosystems [56, 57]. The metals in acid soluble fraction (including the exchangeable and bound to carbonate fractions) are mainly introduced by anthropogenic activities, and are more rapidly bioavailable and easily cause environmental toxicity [58, 59]. The reducible fraction (bound to Fe/Mn oxyhydroxides) can be mobilized when environmental conditions become increasingly reducing, and the oxidizable fraction (bound to organic matter) can be mobilized when environmental conditions become oxidizing [60]. The detrital fraction is composed of metals present in the inert condition, being of lattice origin or primary mineral phases, and can be regarded as a measure of contribution by natural sources [61].

**Fig 4:**
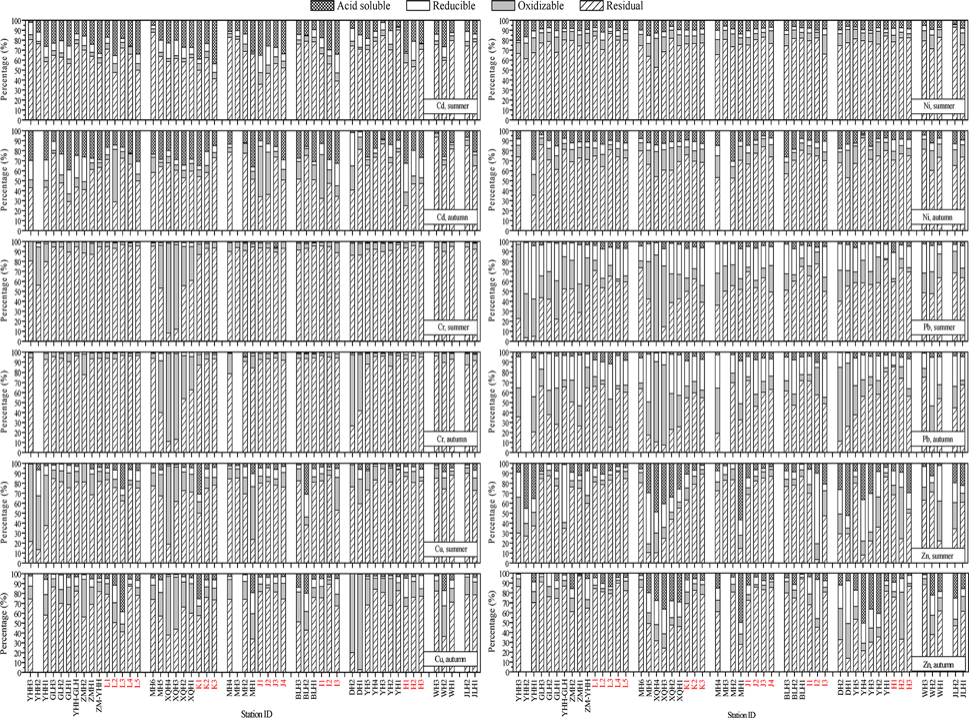
The distributions of the studied metals in different geochemical phases of the surface sediments from the southwestern coastal Laizhou Bay. The data of the samples from the marine sampling sites were indicated with red labels. Related data can be found in S2 Table.

The percentages of heavy metal concentrations that were extracted in each step of the sequential extraction procedure used in this study were presented in Fig. 4. On average, the residual fraction made up the largest proportion of all the studied metals indicating the paramount mineralogical origin of these metals; while differences among the sampling sites were obvious, which might result from the combined effects of the physicochemical conditions of the sedimentary environment, the intensity of anthropogenic activities and so on. Generally, the obviously lower percentages of the studied heavy metals in the residual fraction were most often recorded in the surface sediments of some riverine sampling sites, reflecting that the contribution of anthropogenic sources for the studied heavy metals was more than natural sources at those sites, which further reflected the spatial differences of the impact of various human activities on the local environment. The evidence of this study was consistent with the findings of the research carried out in the coastal area of the Bohai Bay, another heavily anthropogenically influenced major bay in the Bohai Sea, and anthropogenic influence might be responsible for the obvious spatial variations of metal fractionation patterns in riverine sediments [10].

For the riverine sediments, the mean percentages of Cd fractions in both seasons and Zn fractions in summer followed the order of F4 > F1 > F2 > F3, the mean percentages of Cr and Cu fractions in both seasons and Pb fractions in summer followed the order of F4 > F3 > F2 > F1, the mean percentages of Ni fractions in both seasons followed the order of F4 > F3 > F1 > F2, and the mean percentages of Pb and Zn fractions in autumn followed the order of F4 > F2 > F3 > F1.

For the marine sediments, the mean percentages of Cd and Zn fractions in summer followed the order of F4 > F1 > F2 > F3, the mean percentages of Cd and Ni fractions in autumn followed the order of F4 > F1 > F3 > F2, the mean percentages of Cr and Cu fractions in both seasons followed the order of F4 > F3 > F2 > F1, the mean percentages of Ni fractions in summer followed the order of F4 > F3 > F1 > F2, the mean percentages of Pb fractions in both seasons and Zn fractions in autumn followed the order of F4 > F2 > F3 > F1, and the mean percentages of Zn fractions in summer followed the order of F4 > F1 > F2 > F3.

Cd had the highest mean percentage of the acid soluble fraction among the studied metals with the values of 16.3% and 28.6% in summer in the riverine and marine sediments, respectively, and the corresponding values in autumn were 20.7% and 21.8%, respectively. As already mentioned in the previous section, Cd is a typical anthropogenically discharged element and enters the aquatic environment mostly through anthropogenic activities [62]. The percentage of non-residual Cd in this study was much lower than the previous study carried out in the northwestern coastal Bohai Bay [10], which revealed that the overall extent of anthropogenic impact on the distribution of Cd in surface sediments of the southwestern coastal Laizhou Bay was less considerable than that of the northwestern coastal Bohai Bay.

In both seasons, the percentages of the four fractions of Cr and Cu followed the same order F4 > F3 > F2 > F1 in both the riverine and marine sediments. Considering the whole studied area, more than 85% of Cr on average was found in the residual phase in both seasons, while the proportions of the residual fraction for Cu were not that high and the mean values were 71.6% and 67.0% in summer and autumn, respectively. On average, the non-residual Cr and Cu were identified being the highest in the oxidizable fraction in both seasons, and their average percentages in this fraction were clearly higher in the riverine sediments than in the marine sediments. Relatively higher percentages of oxidizable Cr and Cu were recorded at sites XQH3 and XQH4 in both seasons and at sites DH1 and DH2 in autumn. In addition, relatively higher percentages of oxidizable Cu were also recorded in all the three sites in the Yihong River and in MH1 in summer. Generally, the proportions of Cr and Cu in the acid soluble fraction were very low, especially for Cr which was less than 1% of the total concentration in both seasons.

High proportions of residual fraction with a mean value of >70% in both seasons were also observed for Ni. The distribution characteristics of Ni in the four studied geochemical fractions in most sites were similar. Only the site YHH1 showed an obviously higher percentage of non-residual fractions in autumn (64.3%).

Pb had the highest proportion in the reducible fraction among the six studied metals with the mean values of 23.7% and 23.5% in summer in the riverine and the marine sediments, respectively, and the corresponding values in autumn were 26.7% and 22.5%, respectively. Like those of Cr and Cu, the mean percentage of Pb in the oxidizable fraction in the riverine sediments was much higher than that in the marine sediments. This could be explained by the fact that there were more organic matters and sulfide for these metals to combine with and lower redox potential in the riverine sediments than in the marine sediments in the studied area [14].

The mean percentages of Zn in acid soluble fraction in the riverine sediments were much higher than in the marine sediments, which indicated the obvious spatial difference in the anthropogenic impact on the potential mobility of Zn in the studied area. Generally consistent with the spatial distribution characteristics of the studied metals in total concentrations, the relatively higher percentages of Zn in acid soluble fraction were mainly distributed at some sites in the Yihong, the Xiaoqing, the Mi, the Di and the Yu Rivers. A fact that should be noticed is that, although the percentages of Zn in acid soluble fraction in the surface sediments of the sites DH1 and DH2 in autumn were not that high (7.7% and 12.2%, respectively), owing to its high values in total concentration, its concentrations in this fraction were up to 107.2 μg g^-1^ and 1605.4 μg g^-1^, respectively, indicating a very high potential ecological risk.

### Assessment based on sediment quality guidelines

As shown in Fig. 2, the surface sediments for most of the sites were in good condition in both seasons in terms of the concentrations of Cd and Pb, and none of them exceeded the Class II upper limits of the Chinese marine sediment quality standard and PEL values. In the riverine area, the concentrations of Cd exceeded the corresponding TEL value only at four sites, and its concentrations in 10 riverine sediments exceeded the corresponding Class I upper limit. Most of the values were recorded in the Yihong, the Guangli, the Xiaoqing, the Mi, the Yu and the Di Rivers. The condition in autumn was slightly better than in summer. No TEL value and Class I upper limit was exceeded for Cd in any marine sediment. The concentrations of Pb in five riverine sediments exceeded the corresponding TEL value, its concentrations at sites YHH1 (summer) and H1 (summer and autumn) exceeded the corresponding Class I upper limit, and none of them exceeded the TEL value or Class I upper limit in any surface sediment of the rest sites. The condition for Pb in autumn was also better than in summer.

The concentrations of Cr and Ni in the surface sediments of most sites exceeded their corresponding TEL values. The concentrations of Cr at seven riverine sites in summer and six riverine sites in autumn exceeded the corresponding Class I upper limit; they exceeded Class II upper limit in XQH3 and XQH4 in both seasons, and exceeded PEL value at XQH3 in both seasons and at XQH4 in summer. The concentrations of Ni in two riverine sites in summer and four riverine sites in autumn exceeded the corresponding PEL value. Most of the values were also recorded in some of the following rivers: the Yihong River, the Guangli River, the Xiaoqing River, the Mi River, the Yu River and the Di River.

In terms of the concentrations of Cu and Zn, the surface sediments for most of the sites were also in good condition especially in the marine region in both seasons. Except for the concentrations of Cu at the marine sites K3 (summer) and J4 (summer and autumn) which slightly exceeded the corresponding TEL value, the concentrations of Cu and Zn in sediments of all the rest of the marine sites did not exceed their corresponding TEL values and Class I upper limits. The concentration of Cu exceeded both the corresponding PEL value and Class II upper limit at the site YHH2 in summer. The concentrations of Zn exceeded its corresponding PEL value or Class II upper limit at ZMH1, XQH3 and XQH4 in one or both seasons. Extremely high concentrations of Zn were observed at DH1 (1387 μg g^-1^) and DH2 (13213 μg g^-1^) in autumn, which were even much higher than the corresponding value of the Class III upper limit in the Chinese marine sediment quality standard.

**Fig 5:**
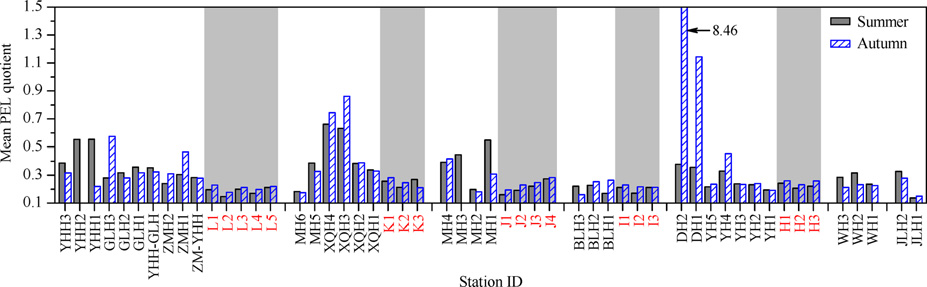
The spatial distribution of mean PEL quotient values in the surface sediments of the studied area. The data of the samples from the marine sampling sites were indicated with light grey background and red labels. Related data can be found in S3 Table.

As shown in Fig. 5, in the surface sediments of the coastal Laizhou Bay, the mean PEL quotients indicated that the combination of the six studied metals had a 21% probability of being toxic in summer at all sampling sites with no exception, and this was also true in autumn for all the studied sites except DH2, where the combination of the six studied metals had a 73% probability of being toxic. This was basically consistent with the indication of the results of acid-volatile sulfide and simultaneously extracted metals analysis conducted for the same samples as in this study [14], and it was also comparable with the findings of the same research carried out in the coastal Bohai Bay [10].

**Fig 6:**
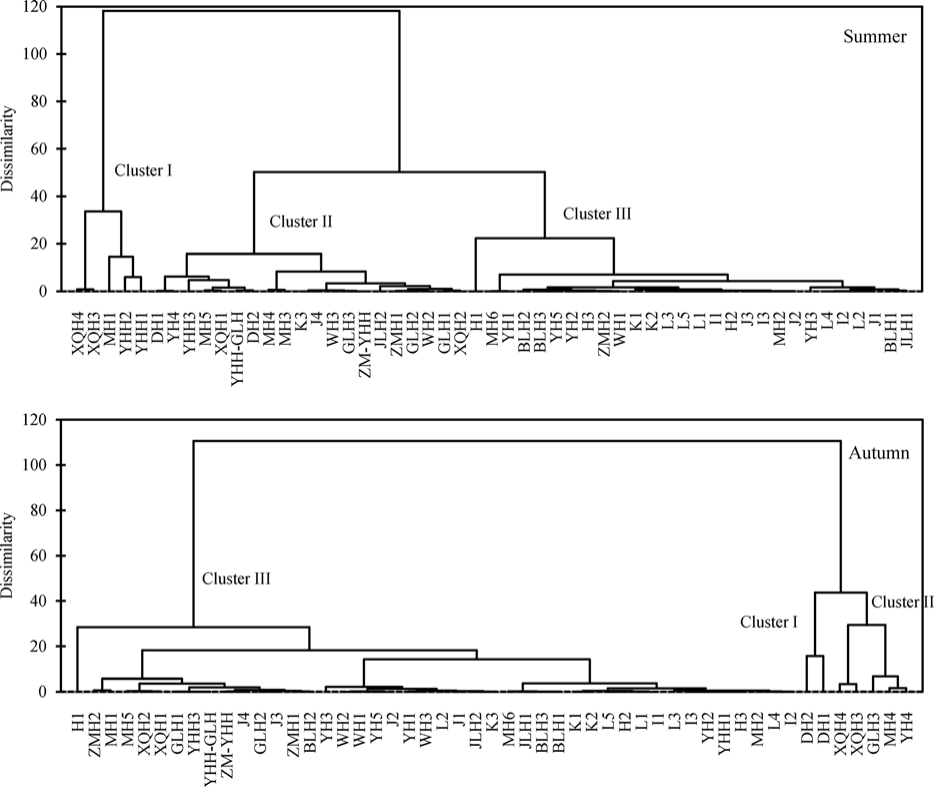
Dendrogram showing clustering of sampling sites in summer and autumn in the southwestern coastal Laizhou Bay.

The result of the AHC analysis of the sampling sites based on the data of metals in total concentrations is shown in Fig. 6. Three main clusters could be clearly observed in both seasons. In summer, Cluster I involved those sites (YHH1, YHH2, XQH3, XQH4 and MH1) that were heavily contaminated according to the SQGs; Cluster II was made up of the sites which were moderately or less contaminated; Cluster III was made up of the remaining sites which were not contaminated, and most sites in the marine region belonged to this cluster. In autumn, Cluster I involved sites DH1 and DH2 which were extremely contaminated by Zn according to the SQGs; Cluster II was made up of the sites (GLH3, XQH3, XQH4, MH4 and YH4) which were heavily contaminated by certain heavy metals according to the SQGs; Cluster III was made up of the remaining sites which were less or not contaminated.

### Assessment based on sediment quality indices

#### 1. Geoaccumulation index

**Fig 7:**
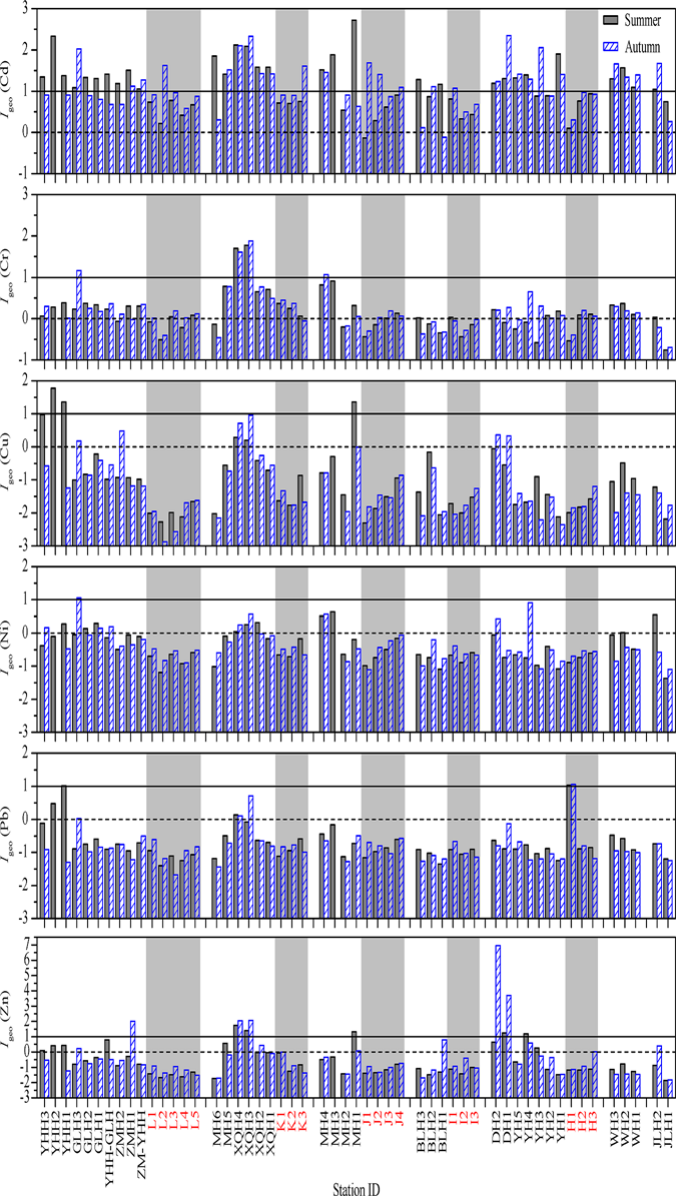
The spatial distributions of *I*_geo_ values for heavy metals in the surface sediments of the studied area. The horizontal dash and solid lines represent *I*_geo_ values of 0 and 1, respectively. The data of the samples from the marine sampling sites were indicated with light grey background and red labels. Related data can be found in S4 Table.

*I*_geo_ is a normalization technique that has been widely applied to the assessment of heavy metal contamination in soils and sediments [23, 27, 28, 39]. The spatial distributions of calculated *I*_geo_ values for the studied metals are shown in Fig. 7. Most of the *I*_geo_ values of Cd in surface sediments of the marine region were between 0 and 1 in both seasons which showed that these sites were in the status of being uncontaminated to moderately contaminated by Cd. *I*_geo_ values of Cd in most of the riverine sediments were between 1 and 2 in one or both seasons, indicating that these sites were moderately contaminated by Cd. The highest *I*_geo_ value of Cd, i.e. 2.7, was recorded in the surface sediment of site MH1 in summer, which showed a moderately to heavily contaminated status.

*I*_geo_ values of Cr at most sites were in the range of 0 to 1, and they exceeded 1 only at GLH3 and MH4 in autumn and XQH3–4 in both seasons, but never exceeded 2. *I*_geo_ values of Cu, Ni, Pb and Zn in the surface sediments of almost all the marine sites were apparently lower than 0, indicating a practically uncontaminated status in both seasons in terms of these heavy metals, and this was true for most of the riverine sites. For the rest of the riverine sites, *I*_geo_ values of Cu, Ni, Pb and Zn fell into the range of 0 to 1 in one or both seasons, and at 10 sites *I*_geo_ values of at least one of these four heavy metals fell into the range of 1 to 2 in one or both seasons. The recorded values with the highest frequency of between 1 and 2 were in the Xiaoqing River, indicating a moderately contaminated status which was obviously caused by human pollution. In autumn, the concentrations of Zn in the surface sediments of DH1 and DH2 reached the heavily and extremely contaminated status reflected by its *I*_geo_ values of 3.7 and 7.0, respectively.

#### 2. Enrichment factor

Like *I*_geo_, EF is also a normalization technique frequently used to separate the metals of natural variability from the fraction that is associated with sediments due to human activities [10, 39]. It is generally considered that an EF value of higher than 1.5 suggests that a significant portion of metals are delivered from non-crustal materials and the sources are likely to be anthropogenic.

**Fig 8:**
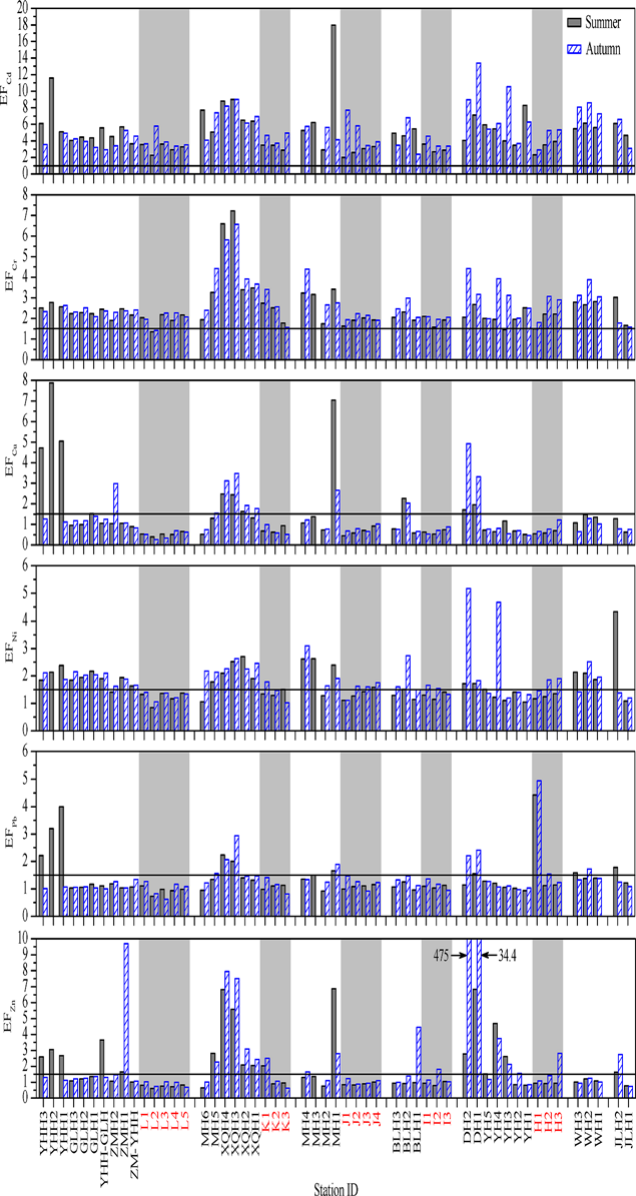
The spatial distributions of EF values for heavy metals in the surface sediments of the studied area. The horizontal lines represent an EF value of 1.5. The data of the samples from the marine sampling sites were indicated with light grey background and red labels. Related data can be found in S5 Table.

The spatial distributions of calculated EFs for each of the studied metals are shown in Fig. 8. The EF values of Cd and Cr in almost all the surface sediments of the coastal Laizhou Bay were higher than 1.5, suggesting the accumulation of anthropogenic inputs. The mean EF values of Cd were 5.0 in summer and 5.4 in autumn which showed a moderate enrichment of Cd in the whole area. The highest EF values of Cd were recorded at site MH1 (18.0) in summer indicating severe enrichment of Cd at this site. The scatter graph for Cd concentrations in summer and autumn showed that Cd in this area was mainly from anthropogenic inputs (Fig. 3), and the EF analysis proved this point. The EF values of Cr at most of the sites reflected a status of minor enrichment, and moderate to moderately severe enrichment of Cr was mainly recorded at the sites in the Xiaoqing River and the Mi River.

In both seasons, the EF values of Cu, Pb and Zn in almost all the marine sites and most of the riverine sites were <1.5, indicating that these metals mainly came from natural origins; Cu and Pb in the rest of the riverine sediments had different degrees of enrichment. Significant portion of Cu at some sites of the Yihong, the Mi and the Di Rivers were in the status of moderate to moderately severe enrichment, and moderate enrichment of Pb at some sites of the Yihong River was recorded. Besides, the EF values reflected that Pb in the surface sediment of the marine site H1 was moderately enriched in both seasons. Zn was moderately-severely enriched at the riverine sites ZMH1, XQH3–4 and MH1, very severely enriched at the riverine site DH1, and extremely severely enriched at the riverine site DH2.

The EF values of Ni indicated that at most of the marine sites especially in summer it was mainly from natural origins, and at most of the riverine sites it was in the status of minor enrichment; its EF value at site DH2 in autumn was slightly higher than the lower limit being used to classify moderately severe enrichment.

#### 3. Risk assessment code

**Fig 9:**
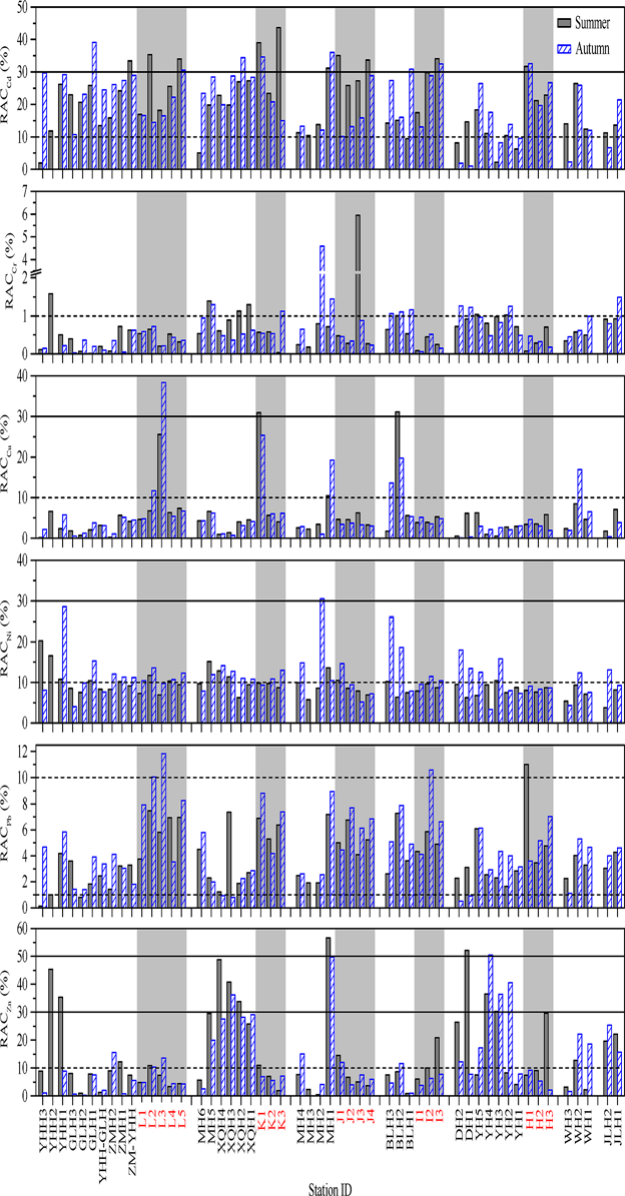
The spatial distributions of RAC values for heavy metals in the surface sediments of the studied area. The horizontal dash lines represent RAC values of either 1% or 10%. The horizontal solid lines represent RAC values of either 30% or 50%. The data of the samples from the marine sampling sites were indicated with light grey background and red labels. Related data can be found in S2 Table.

The spatial distributions of calculated RACs for each of the studied metals were shown in Fig. 9. Although the total concentrations of Cd in the surface sediments of the coastal Laizhou Bay were very low, the proportions of acid soluble fraction of Cd were very high. It showed that most of the sites suffered from medium risk from Cd (10% < RAC ≤ 30%). The highest RAC value of Cd was recorded at site K3 (43.6%) in summer which meant a high risk from Cd.

Most of the sites suffered no risk from Cr (RAC ≤ 1%) and no RAC value exceeded 10%. The highest RAC value of Cr was recorded at site J3 in summer, but the value was only 6%. Most RAC values of Cu and Pb were between 1% and 10%, indicating a low risk from them; the highest values of both Cu (38.3%) and Pb (11.8%) were recorded at L3 in autumn.

The majority of RAC values of Ni fluctuated around 10%, indicating a status of low risk to medium risk. The relatively higher RAC values of Ni were recorded at sites YHH1 (28.6%) and MH2 (30.5%) in summer, indicating a status of medium risk and high risk, respectively.

Most RAC values of Zn in the riverine sediments were between 10% and 30% indicating a medium risk. The relatively higher RAC values of Zn were distributed at the sites in the Yihong, the Xiaoqing, the Mi, the Di and the Yu Rivers. The highest RAC value of Zn was recorded at MH1 (56.6%) in summer which showed a very high risk. Compared with the other marine sites, H3 in summer had the highest RAC value of Zn (29.6%), and the risk level was medium.

It is worth noting that although the acid soluble fraction of Cd occupied considerable proportions of the total contents, the total concentrations of Cd in most sites were very low. The TEL value or the Class I value of Cd was exceeded in a few riverine sites, so the ecological risk of Cd might not be that serious. Similarly, percentage composition of acid soluble Zn was not that high as Cd, but the total concentrations of Zn in surface sediments of some sites were extremely high, especially in DH1 and DH2 in autumn (Fig. 2), they still showed very high ecological risk. Therefore, the ecological risk level of a certain heavy metal depends on the total content and the percentage of acid soluble fractions.

According to the three sediment quality indices above, ecological risk in the riverine sediments was significantly higher than in the adjacent marine area, and the relatively higher risk was recorded in some sites of the Yihong, the Guangli, the Xiaoqing, the Mi, the Yu and the Di Rivers. All indices indicated that Cd showed the highest ecological risk in the studied area especially in the riverine sediment, and Pb showed the lowest ecological risk, and the ecological risks of Cu, Ni and Zn were between them. There was a high consistency between *I*_geo_ and EF of Cr, from which it can be concluded that Cr has obvious enrichment and ecological risk. However, RAC showed there was little possibility of contamination caused by Cr. EF and *I*_geo_ indices were calculated mainly based on the total concentration and the back ground value of a given metal, whereas RAC was based on the chemical form of a given metal neglecting its total concentration. So the different basic principle of these indices might lead to the differences in evaluation results. It is generally accepted that the fractionation can give more information on the bio-availability and bio-toxicity of a certain metal than the total concentration, but it is also difficult to produce any ecological risk if bio-availability content cannot meet the threshold value, so the total content is also very important. Therefore, a variety of evaluation methods should be used to reflect the real situation.

### Sources of heavy metals

PCA can be used to reduce data and extract a smaller number of independent factors (principal components) for analyzing the relationships among observed variables. This method has been widely applied to the identification of pollution sources and the apportionment of natural versus anthropogenic contributions [28, 63, 64]. Table 5 shows that there were two PCs for the surface sediments in the studied area in both seasons. The results of Kaiser-Meyer-Olkin (KMO) for the data of summer and autumn were 0.663 and 0.759, respectively; significances of the Bartlett’s sphericity tests were <0.001 for both the two data sets. So, the data met the conditions of the principal component analysis. These PCs were the ones with eigenvalues larger than 1 and more than 5% of variance could be explained by each PC; altogether they accounted for 67.2% and 65.6% of the variance in the data in summer and autumn, respectively.

**Table 5:** The rotated component matrixes of metals and TOC in the studied area, the Liaodong Bay and the southern Bohai Bay. Bold values indicate strong loadings.

	The SW coastal Laizhou Bay				The Liaodong Bay [42]		The southern Bohai Bay [40]		
	Summer		Autumn						
	PC1	PC2	PC1	PC2	PC1	PC2	PC1	PC2	PC3
Cd	**0.837**	0.039	**0.649**	0.268	-	-	0.34	**0.72**	**0.49**
Cr	**0.660**	**0.505**	**0.785**	**0.495**	**0.93**	0.18	**0.54**	**0.74**	-0.14
Cu	**0.811**	-0.006	**0.687**	**0.511**	**0.75**	0.45	**0.82**	**0.48**	0.25
Ni	**0.468**	**0.694**	**0.804**	0.099	**0.90**	0.35	**0.85**	**0.43**	0.26
Pb	**0.531**	0.170	**0.511**	0.286	0.35	**0.81**	0.31	0.06	**0.90**
Zn	**0.825**	0.039	**0.423**	**0.788**	**0.92**	0.23	**0.84**	0.37	0.26
Al	-0.223	**0.881**	**0.823**	-0.304	**0.83**	0.12	**0.84**	**0.40**	0.32
Fe	0.255	**0.915**	**0.883**	-0.022	**0.89**	0.36	**0.85**	**0.40**	0.30
TOC	**0.668**	0.363	-0.141	**0.669**	**0.73**	0.13	**0.85**	0.36	0.28
Eigenvalue	3.536	3.536	4.072	1.831	6.24	2.69	6.86	2.37	1.73
Total variance (%)	39.291	27.925	45.243	20.343	52.00	22.42	57.2	19.7	14.4
Cumulative variance (%)	39.291	67.216	45.243	65.586	52.00	74.43	57.2	76.9	91.3

In summer, PC1 which explained 39.3% of the total variance was significantly and positively related to all the heavy metals and TOC, but was not significantly related to Al and Fe which are indicators of terrigenous detrital sources [46, 65]. EF values indicated that significant portions of metals especially Cd and Cr were delivered from anthropogenic sources in summer (Fig. 8). The high loading of TOC with PC1 highlighted the influence of organic matter on the distributions of heavy metals in the sediments of the Laizhou Bay in summer. Thus PC1 represented anthropogenic and biogenic sources, and heavy metals in this PC may have originated from similar pollution sources, which is consistent with the conclusion from previous Pearson correlation analysis (Table 4). PC2, which explained 27.9% of the total variance, was significantly and positively related to Cr, Ni, Al and Fe. In addition, most of Cr and Ni were in detrital fractions (Fig. 4). Therefore, PC2 represented sources from terrigenous detrital.

In autumn, PC1 which explained 45.2% of the total variance was significantly and positively related to all the heavy metals and Al/Fe. EF values also indicated that significant portions of metals were delivered from anthropogenic sources in autumn (Fig. 8). Thus PC1 represented terrigenous input including anthropogenic sources and terrigenous detrital sources. PC2 which explained 20.3% of the total variance was significantly and positively related to Cr, Cu, Zn and TOC. Pearson correlation analysis also showed that Cu and Zn had significant correlation with TOC (Table 4). Thus parts of these metals might have been introduced by biogenic activities.

For the comparison purpose, PCs for surface sediments in the Liaodong Bay and the southern Bohai Bay (hereinafter referred to as the Bohai Bay for short) were also shown in Table 5 [40, 42]. It showed that Cr and Ni were mainly from natural sources in all the three bays of the Bohai Sea. In the Laizhou Bay and the Bohai Bay, sources of Cd could be both natural and anthropogenic, though there was no data available for Cd in the Liaodong Bay. But unlike the Laizhou Bay, Pb in surface sediments of the Liaodong Bay and the Bohai Bay displayed no strong correlations with the other metals, suggesting that Pb had another different source or pathway (i.e., aerosol precipitation) [40]. In addition, all the studied metals in the Laizhou Bay (especially in summer) and Cd, Cr, Cu and Ni in the Bohai Bay were partly from anthropogenic sources, whereas most of the studied metals were mainly derived from parent rocks except Pb in the Liaodong Bay. The comparison results indicated there were large differences in natural and man-made environments between different regions of the Bohai Sea.

Considering all the evidence, the overall situation about geochemical characteristics of heavy metals in the surface sediments of the studied area might be as follows. The sampling time in summer was before the peak period of the rainy season, so only a small amount of heavy metals from natural rock weathering sources could be carried into the aquatic environments by rainwater. And the main sources were anthropogenic sources such as effluent and sewage runoff, traffic activities, the burning of fossil fuels and boat maintenance and repair, and/or biogenic sources such as deposition of biological debris. The sampling time in autumn was after the peak period of the rainy season, so a large amount of heavy metals from natural rock weathering sources might have been brought into the rivers and the marine region by rainwater. Therefore, anthropogenic sources, terrigenous detrital sources and biogenic sources all made important contributions to the geochemical characteristics of heavy metals in the sediments in autumn.

## Conclusions

In the surface sediments of the southwestern coastal Laizhou Bay, the total concentrations of heavy metals in the riverine sediments were much higher than in the marine sediments, and the relatively higher concentrations of metals were detected at some sites of the Yihong, the Guangli, the Xiaoqing, the Mi, the Di and the Yu Rivers.

It was found that all the studied metals mainly existed in residual fractions, yet spatial differences were very significant and non-residual fractions dominated their fractionations at some riverine sites. Although Cr had the lowest percentage in acid soluble fraction, the most labile fraction, with the mean value of less than 1% in both seasons, it was obviously enriched at most of the sites; meanwhile, as the most enriched heavy metal, Cd had the highest percentages in the acid soluble fraction among the studied metals with the mean values of 20.5% and 21.1% in summer and autumn, respectively, while its low total concentrations made it hard to cause negative effects to the environment according to the sediment quality guidelines.

Cr, Cu, Ni and Zn were the main pollutants in the riverine sediments according to the sediment quality guidelines. The quality of the marine sediments was generally in good condition, and there was no significant increase in the concentrations of the studied metals compared with the previous studies in surface sediments of the Laizhou Bay. The heavy metals obviously had different sources in different seasons. In summer, the metals were mainly from anthropogenic and biogenic sources; in autumn, the anthropogenic activities and terrigenous detrital were the main sources. This study indicated that, up to now, there was no clear sign that heavy metal pollution was correlated with the ecological degradation of the Laizhou Bay.

## Supporting Information

S1 Table Measured heavy metal data. (PDF)

S2 Table Percentages of metals in different fractions. F1: acid soluble fraction; F2: reducible fraction; F3: oxidizable fraction; F4: Residual fraction.
(PDF)

S3 Table Mean PEL quotient data. (PDF)

S4 Table *I*_geo_ data. (PDF)

S5 Table EF data. (PDF)
