# Supplementary material for: Sediment Quality of the SW Coastal Laizhou Bay, Bohai Sea, China: A Comprehensive Assessment Based on the Analysis of Heavy Metals
Source: PLoS One. 2015 Mar 27;10(3):e0122190. doi: 10.1371/journal.pone.0122190 (PMC4376849; doi:10.1371/journal.pone.0122190)
Supplement: S1 Table — (PDF) [file pone.0122190.s001.pdf]

S1 Table. Measured heavy metal data.

Site	Cd (μg g^{-1})		Cr (μg g^{-1})		Cu (μg g^{-1})		Ni (μg g^{-1})		Pb (μg g^{-1})		Zn (μg g^{-1})	
	May-Jun.	Sept.-Oct.	May-Jun.	Sept.-Oct.	May-Jun.	Sept.-Oct.	May-Jun.	Sept.-Oct.	May-Jun.	Sept.-Oct.	May-Jun.	Sept.-Oct.
YHH3	0.37	0.28	54.9	64.7	73.6	25.1	23.0	33.5	27.7	16.0	115	73.2
YHH2	0.74		63.7		129		27.9		41.8		142	
YHH1	0.38	0.28	68.5	52.7	95.9	15.9	36.3	21.5	60.7	12.2	144	45.3
GLH3	0.31	0.60	61.6	117	18.7	42.4	29.0	62.0	16.2	30.4	61.0	124
GLH2	0.37	0.27	67.8	62.4	21.0	20.7	33.0	28.7	17.8	15.2	72.0	62.5
GLH1	0.36	0.26	66.3	59.2	32.2	28.3	36.8	33.1	19.9	16.7	82.2	78.0
YHH-GLH	0.39	0.23	61.5	67.4	18.9	25.6	27.2	34.2	16.0	16.3	186	75.1
ZMH2	0.33	0.24	50.2	56.6	19.7	52.3	21.2	22.8	17.9	17.7	56.9	73.0
ZMH1	0.42	0.32	64.8	51.6	19.5	16.5	28.9	23.4	15.5	12.8	87.5	429
ZM-YHH	0.31	0.35	64.8	66.6	19.0	16.4	28.0	26.1	18.3	21.2	61.2	60.0
L1	0.24	0.28	49.7	53.0	9.3	9.6	18.5	21.6	15.6	19.6	39.9	56.2
L2	0.17	0.45	36.8	39.7	7.7	5.1	13.1	16.9	11.3	13.2	33.5	41.4
L3	0.25	0.29	54.1	59.7	9.5	6.3	19.2	20.7	13.9	9.4	37.9	54.8
L4	0.20	0.22	45.3	53.2	8.6	11.6	15.8	16.1	12.7	15.6	34.7	47.0
L5	0.23	0.27	55.5	57.0	11.9	12.2	20.0	20.9	14.3	16.9	42.8	37.3
MH6	0.53	0.18	47.8	38.2	9.2	8.4	14.9	19.8	13.2	11.1	32.1	32.5
MH5	0.39	0.42	90.3	89.9	25.5	22.5	28.1	24.8	21.2	18.2	158	94.3
XQH4	0.64	0.63	170	159	45.7	61.2	30.9	35.5	33.0	32.3	357	442
XQH3	0.62	0.74	179	192	43.1	72.9	35.8	44.2	28.3	49.2	281	446
XQH2	0.44	0.39	82.4	89.3	28.1	31.3	37.4	29.4	19.3	19.1	102	143
XQH1	0.44	0.39	85.8	73.8	22.9	25.5	26.7	28.4	18.5	17.1	102	99.5
K1	0.24	0.28	67.8	71.7	12.1	14.9	19.0	21.4	13.8	16.9	101	106
K2	0.24	0.28	62.3	67.9	11.0	11.0	18.3	22.3	15.6	17.5	44.6	56.9
K3	0.25	0.45	54.8	50.5	20.6	11.7	26.6	19.0	20.0	15.1	60.1	41.2
MH4	0.42	0.40	92.5	110	21.7	21.7	42.7	44.3	22.1	19.1	75.0	84.0
MH3	0.54		98.7		30.5		46.6		26.7		85.9	
MH2	0.21	0.28	45.6	46.4	13.7	9.6	19.2	16.5	13.7	12.4	39.6	39.0
MH1	0.96	0.23	65.5	54.3	96.3	37.3	26.2	21.4	18.1	21.2	267	112
J1	0.13	0.47	38.8	42.6	7.6	10.7	15.1	13.9	13.4	18.5	40.7	54.8
J2	0.18	0.39	47.4	53.5	10.3	13.6	17.9	22.2	15.3	17.3	41.6	42.5
J3	0.23	0.27	52.9	59.8	13.2	12.9	21.3	25.5	16.6	14.6	47.5	53.0
J4	0.28	0.31	57.5	54.9	19.5	20.7	27.0	28.8	19.7	20.2	61.1	64.3
BLH3	0.36	0.16	53.3	40.7	14.5	8.8	19.1	15.1	15.9	12.5	50.1	33.1
BLH2	0.27	0.32	48.0	49.8	33.4	24.2	17.9	26.0	14.8	14.1	38.2	47.2
BLH1	0.33	0.14	41.4	41.8	9.0	9.6	14.1	17.5	11.8	13.1	42.8	185
I1	0.26	0.31	53.7	50.5	11.4	9.1	18.9	23.0	15.9	18.9	48.9	55.5
I2	0.19	0.21	38.7	43.2	9.3	11.0	16.2	19.3	14.4	14.7	39.3	80.7

Site	Cd (μg g^{-1})		Cr (μg g^{-1})		Cu (μg g^{-1})		Ni (μg g^{-1})		Pb (μg g^{-1})		Zn (μg g^{-1})	
	May-Jun.	Sept.-Oct.	May-Jun.	Sept.-Oct.	May-Jun.	Sept.-Oct.	May-Jun.	Sept.-Oct.	May-Jun.	Sept.-Oct.	May-Jun.	Sept.-Oct.
I3	0.20	0.24	47.7	51.6	13.0	15.6	19.9	18.9	16.0	13.6	52.4	51.6
DH2	0.33	0.35	61.2	60.5	36.1	48.3	28.9	40.5	19.4	17.3	167	13214
DH1	0.36	0.75	49.3	63.2	25.5	47.2	18.1	20.8	16.2	27.4	254	1387
YH5	0.37	0.39	44.2	51.4	11.2	14.1	19.0	20.1	16.1	18.7	67.7	61.3
YH4	0.39	0.36	49.5	82.5	11.7	12.0	17.8	56.0	17.5	12.8	241	160
YH3	0.27	0.61	35.2	64.9	20.0	8.1	15.2	14.2	14.6	13.0	128	88.6
YH2	0.27	0.27	55.4	52.9	13.8	13.0	22.7	21.0	16.3	14.6	48.8	82.5
YH1	0.55	0.39	59.5	55.2	8.6	7.3	14.2	16.6	12.6	13.1	38.2	38.5
H1	0.16	0.18	36.2	39.9	9.4	10.4	16.2	18.5	61.5	62.6	46.7	48.8
H2	0.25	0.29	55.9	60.4	10.6	10.7	18.0	20.8	16.2	17.3	47.0	56.1
H3	0.28	0.28	56.5	54.6	12.5	16.3	19.7	20.4	16.6	13.2	49.1	107
WH3	0.36	0.46	66.1	64.3	18.1	9.4	28.8	16.6	21.5	15.5	48.5	38.5
WH2	0.43	0.37	67.6	59.8	26.7	14.2	30.3	22.2	20.0	15.3	61.6	39.1
WH1	0.31	0.39	56.3	57.7	19.3	13.7	21.3	21.2	15.9	15.0	44.4	38.6
JLH2	0.30	0.47	53.7	45.3	16.1	14.2	43.7	20.1	18.0	18.0	58.7	141
JLH1	0.25	0.18	31.0	32.4	8.2	11.0	11.6	14.0	13.1	12.7	29.2	30.2
