# Supplementary material for: Sediment Quality of the SW Coastal Laizhou Bay, Bohai Sea, China: A Comprehensive Assessment Based on the Analysis of Heavy Metals
Source: PLoS One. 2015 Mar 27;10(3):e0122190. doi: 10.1371/journal.pone.0122190 (PMC4376849; doi:10.1371/journal.pone.0122190)
Supplement: S2 Table — F1: acid soluble fraction; F2: reducible fraction; F3: oxidizable fraction; F4: Residual fraction. (PDF) [file pone.0122190.s002.pdf]

S2 Table. Percentages of metals in different fractions. F1: acid soluble fraction; F2: reducible fraction; F3: oxidizable fraction; F4: Residual fraction.

Site	Cd (%)								Cr (%)								Cu (%)							
	May-Jun.				Sept.-Oct.				May-Jun.				Sept.-Oct.				May-Jun.				Sept.-Oct.			
	F1	F2	F3	F4	F1	F2	F3	F4	F1	F2	F3	F4	F1	F2	F3	F4	F1	F2	F3	F4	F1	F2	F3	F4
YHH3	1.92	12.59	4.82	80.67	29.70	19.25	7.95	43.11	0.11	0.03	19.26	80.60	0.15	0.73	5.05	94.07	0.11	0.59	77.75	21.54	2.16	10.46	13.09	74.30
YHH2	11.86	9.39	2.31	76.45					1.58	3.97	38.20	56.25					6.59	26.04	54.03	13.35				
YHH1	26.16	11.03	4.29	58.51	29.11	20.66	6.78	43.46	0.50	1.73	17.95	79.81	0.22	1.34	6.03	92.42	2.36	9.69	50.32	37.64	5.78	15.32	20.59	58.32
GLH3	22.93	7.51	4.83	64.73	10.67	9.14	2.68	77.52	0.40	0.40	4.63	94.57	0.03	0.46	4.13	95.38	1.77	5.15	8.53	84.55	0.47	4.67	8.24	86.63
GLH2	20.62	12.24	4.11	63.03	23.13	20.96	8.15	47.77	0.07	0.50	4.89	94.54	0.36	0.98	5.05	93.61	0.69	7.07	10.77	81.47	1.22	14.85	13.80	70.12
GLH1	25.82	13.23	4.26	56.69	39.08	23.95	7.87	29.10	0.01	0.86	9.79	89.33	0.20	1.09	8.46	90.26	2.01	7.81	14.82	75.37	3.74	9.87	17.51	68.87
YHH-GLH	13.47	6.10	3.96	76.47	24.46	22.65	9.16	43.73	0.20	0.19	4.98	94.64	0.10	1.04	2.71	96.15	3.16	5.19	10.30	81.36	3.10	9.40	5.16	82.34
ZMH2	15.81	9.62	4.59	69.98	26.10	24.91	7.66	41.33	0.07	0.69	10.81	88.43	0.35	1.86	19.88	77.91	0.18	0.21	18.30	81.31	1.04	4.57	37.98	56.40
ZMH1	24.15	8.01	3.92	63.92	27.36	4.74	6.53	61.37	0.72	1.26	11.03	86.99	0.05	0.81	6.07	93.08	5.60	5.18	20.70	68.52	5.16	8.35	17.46	69.04
ZM-YHH	33.35	4.30	5.26	57.10	28.93	3.04	4.59	63.43	0.63	0.13	4.54	94.71	0.62	0.19	5.61	93.58	4.14	4.16	9.96	81.75	4.44	3.84	9.71	82.02
L1	16.93	6.16	5.99	70.92	16.54	7.37	4.77	71.32	0.54	0.31	4.39	94.76	0.59	0.42	5.41	93.59	4.66	4.94	6.95	83.46	4.77	5.38	10.64	79.22
L2	35.33	8.23	8.70	47.74	14.43	4.37	52.25	28.96	0.65	0.81	5.45	93.09	0.72	0.87	5.49	92.91	6.75	9.47	8.32	75.47	11.69	17.60	20.07	50.64
L3	18.15	3.91	5.84	72.10	16.48	4.53	6.48	72.51	0.21	0.45	2.70	96.65	0.21	0.48	3.01	96.30	25.54	6.64	5.60	62.23	38.32	12.66	7.81	41.21
L4	25.59	0.92	7.61	65.88	22.21	0.80	10.43	66.56	0.52	0.91	3.30	95.27	0.44	0.85	2.97	95.74	6.30	10.93	4.47	78.30	5.41	5.23	2.06	87.31
L5	33.97	9.35	6.64	50.04	30.54	12.78	6.76	49.93	0.31	0.52	3.72	95.45	0.36	0.55	2.65	96.44	7.33	10.76	6.86	75.04	6.65	7.42	7.86	78.08
MH6	5.03	3.76	3.16	88.05	23.42	3.09	15.15	58.34	0.54	0.85	3.46	95.16	0.95	0.62	4.81	93.62	4.28	5.42	12.93	77.38	4.26	7.41	14.49	73.84
MH5	19.81	12.47	4.06	63.67	28.40	3.12	4.41	64.07	1.39	2.35	42.99	53.27	1.29	7.60	51.08	40.02	6.63	4.73	21.35	67.29	6.16	12.83	23.63	57.38
XQH4	22.81	15.55	3.11	58.53	19.84	5.83	6.50	67.83	0.61	1.09	90.01	8.30	0.47	0.91	87.62	11.00	0.96	1.60	78.60	18.84	1.04	1.70	59.21	38.05
XQH3	19.81	15.86	2.70	61.63	28.76	5.33	5.39	60.52	0.89	2.18	85.03	11.91	0.36	1.40	85.17	13.08	1.34	2.40	34.63	61.63	0.70	2.82	52.57	43.90
XQH2	26.98	11.21	3.64	58.17	34.40	5.16	7.30	53.14	1.13	3.84	39.47	55.57	0.53	3.31	42.27	53.90	4.03	4.86	18.54	72.58	3.06	5.45	24.96	66.53
XQH1	27.31	6.79	3.48	62.41	28.38	4.25	7.80	59.57	1.30	3.98	33.72	61.01	0.62	4.05	32.48	62.86	4.51	6.61	17.94	70.94	4.08	6.09	29.73	60.10
K1	39.00	4.50	6.50	50.00	34.63	4.46	6.75	54.16	0.57	2.21	10.33	86.89	0.54	2.18	10.40	86.88	30.90	7.77	11.32	50.00	25.36	7.15	9.85	57.64
K2	23.43	7.85	6.14	62.58	20.82	13.95	6.95	58.28	0.58	1.46	4.89	93.07	0.54	1.42	5.24	92.81	5.61	8.96	10.67	74.76	6.00	7.33	10.77	75.90

Site	Cd (%)								Cr (%)								Cu (%)							
	May-Jun.				Sept.-Oct.				May-Jun.				Sept.-Oct.				May-Jun.				Sept.-Oct.			
	F1	F2	F3	F4	F1	F2	F3	F4	F1	F2	F3	F4	F1	F2	F3	F4	F1	F2	F3	F4	F1	F2	F3	F4
K3	43.63	8.78	6.27	41.32	14.97	6.82	5.18	73.04	0.03	0.69	5.75	93.53	1.12	1.50	4.31	93.07	4.04	10.14	7.80	78.02	6.11	8.48	11.82	73.59
MH4	11.26	2.13	3.80	82.81	13.33	3.46	4.83	78.39	0.24	0.91	9.00	89.85	0.65	0.96	19.67	78.72	2.52	2.91	10.14	84.43	2.83	3.08	20.02	74.07
MH3	10.35	5.83	2.78	81.05					0.18	0.42	5.58	93.82					2.23	3.70	9.01	85.06				
MH2	13.74	10.21	7.32	68.74	12.07	0.62	9.91	77.40	0.79	1.10	7.22	90.89	4.58	0.48	4.45	90.49	3.39	11.00	16.32	69.29	0.97	6.93	18.08	74.02
MH1	31.17	1.19	1.63	66.02	36.02	4.58	7.84	51.56	0.71	1.25	3.65	94.39	1.45	2.43	12.21	83.91	10.42	13.06	52.85	23.67	19.21	21.23	25.50	34.06
J1	35.07	17.42	11.72	35.79	10.16	5.42	50.43	34.00	0.47	1.11	4.91	93.51	0.45	1.08	5.98	92.48	4.64	8.35	6.89	80.11	3.42	7.08	7.54	81.97
J2	25.78	19.32	8.08	46.82	13.12	8.43	42.07	36.39	0.28	1.48	5.04	93.21	0.34	1.33	5.02	93.31	4.55	9.28	4.59	81.58	3.72	7.61	2.98	85.70
J3	27.28	9.54	6.62	56.56	15.87	4.88	5.43	73.82	5.95	0.95	3.62	89.48	0.88	0.59	4.54	93.99	6.25	9.56	6.19	78.00	3.30	6.36	8.86	81.48
J4	33.72	7.29	6.91	52.08	28.84	9.83	10.57	50.77	0.27	0.22	6.45	93.06	0.23	0.40	7.62	91.75	3.25	6.17	14.57	76.01	2.98	6.78	15.85	74.38
BLH3	14.22	5.48	4.29	76.00	27.39	3.33	17.55	51.74	0.64	1.05	6.88	91.43	1.06	0.73	4.60	93.62	1.75	3.92	12.03	82.31	13.59	4.90	30.04	51.48
BLH2	15.06	0.55	5.33	79.06	15.98	2.04	6.47	75.51	1.01	1.10	4.78	93.11	1.10	0.69	4.89	93.32	31.11	22.88	7.85	38.16	19.74	18.49	18.80	42.97
BLH1	9.42	7.78	4.31	78.49	30.86	3.36	14.78	51.00	0.53	0.82	3.23	95.41	1.16	0.92	4.00	93.92	5.54	8.27	5.23	80.96	5.31	8.72	9.98	75.99
I1	17.44	10.35	5.90	66.31	13.02	10.72	44.02	32.24	0.08	0.73	3.59	95.60	0.06	0.87	2.54	96.54	3.83	6.79	7.12	82.25	5.11	7.87	11.40	75.62
I2	29.79	6.04	8.04	56.14	28.80	10.21	13.53	47.46	0.45	0.05	4.68	94.82	0.52	0.11	4.42	94.96	3.89	3.04	5.08	88.00	3.54	2.63	4.51	89.32
I3	34.09	18.68	8.21	39.02	32.49	22.53	10.48	34.50	0.25	1.20	9.77	88.77	0.15	1.65	4.20	94.01	5.23	8.94	32.90	52.93	4.82	16.03	11.48	67.67
DH2	8.10	12.96	4.70	74.24	1.86	33.21	24.08	40.85	0.72	1.13	11.90	86.26	1.26	1.89	70.28	26.57	0.51	1.34	22.02	76.13	0.09	0.10	79.62	20.20
DH1	14.59	12.41	1.97	71.03	0.97	5.57	12.14	81.32	0.92	1.38	11.35	86.35	1.22	1.49	55.45	41.83	6.14	9.83	24.76	59.27	0.34	0.08	96.97	2.61
YH5	18.28	6.82	4.17	70.74	26.41	2.42	5.76	65.42	1.04	0.86	5.78	92.32	0.97	0.44	10.86	87.73	6.25	1.16	19.52	73.07	2.88	2.29	26.28	68.55
YH4	11.08	6.13	3.85	78.94	17.57	4.99	3.97	73.47	0.81	0.36	6.77	92.06	0.48	0.70	3.39	95.44	0.94	2.02	13.78	83.27	2.13	2.33	11.23	84.30
YH3	2.19	7.53	5.72	84.56	8.19	0.90	4.16	86.75	0.98	1.06	8.23	89.73	0.83	0.55	3.00	95.62	0.48	0.12	5.04	94.37	2.61	2.06	14.15	81.18
YH2	10.36	13.72	5.62	70.30	13.82	12.78	5.25	68.16	1.03	0.55	7.37	91.05	1.25	1.61	11.48	85.65	2.75	2.09	13.95	81.21	2.04	2.93	27.13	67.90
YH1	6.29	0.76	2.71	90.24	9.53	8.25	3.67	78.55	0.72	0.60	1.93	96.75	0.49	0.56	2.79	96.16	2.89	3.03	0.71	93.37	3.03	6.11	8.07	82.80
H1	31.66	1.39	9.61	57.33	32.56	28.81	13.65	24.98	0.08	1.32	5.15	93.45	0.47	2.09	4.28	93.16	3.39	5.94	4.12	86.56	4.56	19.61	8.73	67.10

Site	Cd (%)								Cr (%)								Cu (%)							
	May-Jun.				Sept.-Oct.				May-Jun.				Sept.-Oct.				May-Jun.				Sept.-Oct.			
	F1	F2	F3	F4	F1	F2	F3	F4	F1	F2	F3	F4	F1	F2	F3	F4	F1	F2	F3	F4	F1	F2	F3	F4
H2	21.15	19.12	6.49	53.24	19.63	25.62	7.92	46.83	0.28	0.37	3.58	95.77	0.33	0.41	3.16	96.10	3.50	4.87	10.92	80.71	2.98	4.92	15.85	76.26
H3	22.90	0.87	5.38	70.84	26.68	20.21	5.98	47.13	0.71	0.92	3.37	95.00	0.18	1.38	3.55	94.90	5.78	8.26	3.83	82.13	1.90	13.36	7.58	77.16
WH3	13.96	6.70	3.88	75.47	2.23	0.31	3.96	93.50	0.34	0.46	5.32	93.88	0.45	0.28	3.32	95.96	2.37	3.03	9.69	84.91	1.92	5.27	10.25	82.57
WH2	26.43	10.96	2.96	59.65	25.86	3.36	5.04	65.75	0.57	0.74	8.64	90.04	0.62	0.91	9.10	89.38	8.46	6.20	11.06	74.28	16.91	15.46	31.15	36.48
WH1	12.33	3.33	4.57	79.78	11.99	2.04	4.31	81.67	0.50	0.02	4.63	94.86	0.99	0.50	5.77	92.74	4.63	3.22	4.29	87.86	6.47	8.48	13.84	71.21
JLH2	11.22	10.02	4.58	74.18	6.68	2.96	5.26	85.10	0.91	0.01	4.56	94.52	0.79	0.87	11.36	86.99	1.71	3.02	5.42	89.84	0.38	3.01	17.79	78.82
JLH1	13.64	4.96	3.13	78.28	21.40	2.89	10.83	64.88	0.93	0.74	6.03	92.30	1.49	0.94	6.13	91.44	7.03	7.82	12.46	72.69	3.84	4.41	12.98	78.77

S2 Table. Continued.

Site	Ni (%)								Pb (%)								Zn (%)							
	May-Jun.				Sept.-Oct.				May-Jun.				Sept.-Oct.				May-Jun.				Sept.-Oct.			
	F1	F2	F3	F4	F1	F2	F3	F4	F1	F2	F3	F4	F1	F2	F3	F4	F1	F2	F3	F4	F1	F2	F3	F4
YHH3	20.30	2.13	10.76	66.82	8.10	6.50	11.51	73.88	0.14	2.88	74.28	22.71	4.66	30.73	28.95	35.66	8.93	25.11	35.95	30.01	1.10	4.66	7.73	86.52
YHH2	16.57	6.44	15.62	61.38					1.01	51.45	44.02	3.53					45.27	15.20	12.61	26.92				
YHH1	10.76	7.01	14.83	67.40	28.57	15.23	20.51	35.69	4.19	53.36	37.62	4.83	5.83	38.38	35.21	20.58	35.32	14.03	13.42	37.23	8.80	9.97	11.03	70.20
GLH3	8.54	3.94	10.22	77.29	4.02	3.50	6.53	85.96	3.59	30.98	21.77	43.66	1.43	15.85	16.40	66.32	7.99	2.72	4.83	84.46	0.64	3.06	5.29	91.01
GLH2	7.54	4.64	9.08	78.73	9.81	7.95	11.38	70.87	0.81	29.48	27.59	42.12	1.41	36.35	24.33	37.91	0.91	6.28	5.38	87.43	0.22	13.38	10.29	76.11
GLH1	10.48	6.48	15.20	67.85	15.27	9.62	13.72	61.39	1.84	37.60	38.49	22.07	3.92	31.63	36.30	28.15	7.80	7.70	7.19	77.31	7.52	8.06	10.03	74.40
YHH-GLH	8.36	3.08	8.32	80.25	7.63	5.40	5.65	81.33	2.45	12.94	32.10	52.51	3.39	24.04	6.79	65.78	1.27	58.11	5.99	34.64	2.02	5.60	5.63	86.75
ZMH2	8.31	2.98	9.03	79.69	12.04	6.79	12.19	68.98	1.43	17.45	28.74	52.38	4.11	23.69	21.60	50.59	9.00	8.46	6.35	76.19	15.62	9.94	13.36	61.09
ZMH1	10.23	4.42	11.11	74.24	11.29	5.54	10.40	72.77	3.23	39.29	28.75	28.74	3.04	50.26	20.18	26.52	12.21	5.07	7.89	74.83	0.72	0.93	1.22	97.13
ZM-YHH	9.12	4.04	9.71	77.13	11.20	5.46	12.13	71.22	3.29	13.25	27.64	55.82	1.82	13.55	19.92	64.72	7.42	24.99	7.88	59.71	5.54	21.39	8.34	64.73

Site	Ni (%)								Pb (%)								Zn (%)							
	May-Jun.				Sept.-Oct.				May-Jun.				Sept.-Oct.				May-Jun.				Sept.-Oct.			
	F1	F2	F3	F4	F1	F2	F3	F4	F1	F2	F3	F4	F1	F2	F3	F4	F1	F2	F3	F4	F1	F2	F3	F4
L1	7.28	3.76	4.75	84.21	10.42	7.04	7.76	74.77	3.75	15.14	10.09	71.02	7.91	16.27	9.70	66.13	4.76	8.99	4.94	81.32	4.75	7.49	3.53	84.23
L2	11.77	8.04	9.50	70.69	13.59	10.14	12.18	64.09	7.47	29.64	9.26	53.63	10.04	17.88	3.79	68.29	10.89	6.16	4.37	78.59	10.33	5.35	3.24	81.08
L3	6.95	4.38	2.11	86.57	9.76	4.18	5.06	81.00	5.82	18.12	10.79	65.27	11.83	34.87	28.08	25.23	7.39	4.32	5.20	83.09	13.65	3.16	4.11	79.09
L4	10.32	5.53	4.05	80.10	10.72	7.14	8.12	74.02	6.93	30.88	2.14	60.06	3.54	29.57	3.24	63.65	3.35	4.20	1.32	91.14	4.41	2.86	3.02	89.71
L5	9.48	3.92	6.48	80.12	12.29	7.18	7.66	72.87	6.96	27.49	6.18	59.38	8.27	24.21	7.30	60.22	4.43	3.92	7.11	84.54	4.31	3.71	9.76	82.22
MH6	9.71	4.67	11.15	74.47	7.85	4.27	8.06	79.81	4.51	14.96	6.79	73.75	5.78	24.77	5.65	63.81	5.65	3.84	10.12	80.39	2.51	4.06	7.97	85.46
MH5	15.17	8.18	12.75	63.90	11.93	7.80	13.77	66.50	2.30	17.91	37.56	42.23	1.98	47.42	33.27	17.33	29.53	51.33	8.71	10.44	19.88	20.23	10.91	48.98
XQH4	12.87	5.33	29.05	52.74	14.18	6.74	24.88	54.20	1.21	12.52	86.26	0.02	0.94	8.79	79.81	10.45	48.80	21.18	19.89	10.13	27.60	24.47	16.45	31.48
XQH3	11.39	5.32	14.78	68.51	12.71	6.30	20.17	60.82	7.35	17.16	61.03	14.46	0.79	12.21	79.67	7.33	40.72	23.60	11.03	24.65	36.26	25.15	14.38	24.21
XQH2	6.26	3.75	7.98	82.01	11.02	6.13	22.23	60.62	1.88	30.54	28.89	38.70	2.26	38.72	35.92	23.10	33.79	15.41	7.08	43.72	28.10	16.80	7.51	47.60
XQH1	9.46	5.17	10.87	74.50	10.84	5.01	14.84	69.31	2.71	30.36	24.46	42.48	2.85	40.13	31.39	25.63	25.73	13.28	6.04	54.95	29.13	15.16	9.71	46.00
K1	9.86	5.93	7.87	76.35	9.33	7.17	7.55	75.96	6.89	29.82	13.29	50.00	8.82	24.69	11.77	54.72	10.97	13.88	12.01	63.14	6.91	12.27	11.51	69.31
K2	9.78	6.04	7.44	76.74	10.91	9.22	10.25	69.63	5.28	22.09	10.27	62.36	4.18	24.77	11.34	59.71	7.04	6.19	7.73	79.04	5.63	5.06	6.65	82.66
K3	8.74	5.16	8.79	77.31	13.01	5.43	13.17	68.40	6.38	37.69	16.81	39.13	7.38	30.17	7.70	54.75	1.91	4.41	4.46	89.22	7.12	4.63	9.79	78.46
MH4	9.94	9.54	14.98	65.54	14.84	10.02	21.85	53.29	2.46	29.31	32.19	36.04	2.62	32.94	45.31	19.14	7.66	11.03	9.05	72.26	15.03	12.45	11.49	61.03
MH3	5.77	4.43	9.01	80.79					1.92	22.61	25.29	50.18					2.36	9.20	5.56	82.88				
MH2	8.58	5.17	12.82	73.44	30.46	5.35	11.52	52.67	1.91	21.49	20.98	55.63	2.56	18.17	9.37	69.91	0.46	5.45	10.72	83.37	4.06	7.41	7.63	80.89
MH1	13.61	4.05	6.43	75.91	10.43	5.16	13.82	70.60	7.18	30.12	10.91	51.79	8.94	42.33	16.17	32.56	56.56	15.67	13.09	14.69	49.77	11.80	10.50	27.93
J1	10.50	6.81	7.50	75.19	14.66	7.53	11.00	66.81	5.01	20.86	4.55	69.58	4.46	20.06	3.52	71.96	14.49	10.20	6.67	68.64	11.99	9.91	5.54	72.57
J2	8.53	4.84	6.06	80.57	9.45	4.32	8.58	77.64	6.77	32.22	7.46	53.55	7.69	34.00	12.13	46.17	6.68	6.61	4.27	82.45	3.98	7.76	2.38	85.88
J3	7.90	4.35	4.62	83.14	5.19	2.78	8.05	83.98	4.10	22.73	9.44	63.73	6.11	22.89	10.65	60.35	5.02	3.67	6.28	85.04	7.47	4.73	4.67	83.12
J4	6.90	3.65	12.62	76.83	7.26	6.86	11.89	73.99	5.23	19.05	26.43	49.29	6.85	16.94	13.17	63.05	3.69	2.52	6.86	86.93	5.92	2.95	5.64	85.49
BLH3	10.19	3.96	11.46	74.39	26.05	5.51	11.34	57.10	2.60	29.95	16.37	51.08	5.08	23.13	10.15	61.64	7.55	8.64	7.27	76.55	4.63	4.16	11.42	79.79

Site	Ni (%)								Pb (%)								Zn (%)							
	May-Jun.				Sept.-Oct.				May-Jun.				Sept.-Oct.				May-Jun.				Sept.-Oct.			
	F1	F2	F3	F4	F1	F2	F3	F4	F1	F2	F3	F4	F1	F2	F3	F4	F1	F2	F3	F4	F1	F2	F3	F4
BLH2	6.37	3.41	8.52	81.70	18.62	3.40	7.45	70.52	7.26	26.12	6.43	60.19	7.87	33.74	11.08	47.31	8.68	7.28	6.04	78.00	11.62	5.53	8.00	74.85
BLH1	7.47	4.78	5.68	82.07	7.85	4.37	7.44	80.35	3.63	13.32	8.07	74.99	4.91	17.32	5.85	71.92	0.82	5.95	6.57	86.67	0.96	3.36	1.55	94.14
I1	7.93	4.84	7.82	79.41	9.53	5.57	8.11	76.79	4.34	20.28	9.75	65.64	4.11	17.96	6.42	71.52	6.03	15.36	8.69	69.93	3.78	17.55	10.50	68.17
I2	9.68	2.39	5.55	82.38	11.50	5.85	7.90	74.74	5.85	4.71	12.04	77.40	10.58	5.04	26.77	57.62	10.02	70.66	15.94	3.38	6.29	39.61	8.93	45.18
I3	8.70	5.94	19.21	66.16	10.45	10.86	8.56	70.13	4.91	30.91	14.35	49.83	6.63	38.21	6.48	48.68	20.83	6.86	25.00	47.31	7.74	6.58	4.87	80.81
DH2	9.55	3.94	11.94	74.57	17.96	3.43	10.52	68.09	2.28	26.52	31.00	40.20	0.51	14.87	73.43	11.20	26.37	24.63	19.65	29.35	12.15	23.69	31.49	32.66
DH1	6.25	2.94	13.38	77.43	13.43	6.28	27.17	53.13	3.11	26.19	15.41	55.29	0.90	10.31	62.66	26.13	52.20	12.93	5.68	29.19	7.73	43.16	35.11	14.00
YH5	6.79	2.18	9.68	81.35	12.50	2.79	17.45	67.25	6.08	24.49	10.66	58.76	6.12	26.96	29.21	37.71	7.59	5.92	6.38	80.11	17.25	16.28	12.95	53.52
YH4	9.43	3.39	7.67	79.51	3.31	1.11	2.80	92.77	2.54	16.40	22.49	58.57	2.93	24.35	16.47	56.24	36.52	41.46	13.98	8.04	50.45	20.44	7.77	21.35
YH3	10.42	2.60	8.43	78.56	15.82	3.32	9.44	71.42	2.30	27.89	12.94	56.88	4.35	20.13	8.11	67.41	30.31	38.90	8.73	22.05	36.40	19.12	14.13	30.35
YH2	7.48	3.67	12.79	76.06	8.00	3.38	13.86	74.77	1.64	13.79	25.88	58.69	3.99	24.08	13.78	58.15	8.24	25.64	30.09	36.03	40.54	14.41	9.46	35.59
YH1	8.81	3.63	3.29	84.27	7.28	4.56	8.09	80.07	2.83	14.35	1.43	81.39	3.15	12.93	3.48	80.45	4.06	5.52	4.16	86.25	7.82	4.72	5.94	81.52
H1	8.04	7.02	6.54	78.40	9.07	11.55	8.68	70.70	11.01	26.33	2.88	59.78	3.58	9.76	1.59	85.07	7.42	14.68	9.62	68.28	9.17	9.62	5.41	75.79
H2	7.61	4.34	5.71	82.34	8.37	6.51	8.28	76.84	3.45	13.73	9.66	73.15	5.15	9.57	11.01	74.27	9.06	15.10	51.42	24.43	5.32	14.00	47.46	33.22
H3	8.72	4.80	4.23	82.26	8.68	9.04	7.17	75.11	4.76	21.57	4.21	69.47	7.03	29.00	7.57	56.40	29.61	16.52	4.04	49.82	2.07	8.44	2.79	86.70
WH3	5.37	5.26	10.14	79.23	4.35	4.17	10.01	81.47	2.27	29.49	19.65	48.60	1.13	15.34	8.53	75.00	3.18	20.95	12.94	62.92	1.52	4.04	6.33	88.10
WH2	9.37	5.41	13.88	71.35	12.37	7.64	19.63	60.37	4.04	26.35	22.07	47.54	5.29	48.04	28.52	18.15	12.74	6.88	9.23	71.16	22.12	20.45	19.51	37.91
WH1	7.13	2.30	7.81	82.76	7.54	6.03	12.98	73.45	3.28	9.19	24.00	63.53	4.65	27.52	13.98	53.85	2.19	35.90	39.77	22.15	18.58	6.48	13.11	61.84
JLH2	3.78	0.59	2.75	92.89	13.11	6.02	15.55	65.32	3.03	6.85	21.61	68.51	3.99	29.77	21.52	44.72	19.64	19.74	26.25	34.37	25.28	21.18	6.76	46.79
JLH1	8.18	4.63	11.85	75.34	9.30	4.99	11.95	73.76	4.26	22.56	9.48	63.70	4.61	23.33	4.37	67.69	22.19	7.97	8.98	60.87	15.64	7.98	15.00	61.38
