# Supplementary material for: Sediment Quality of the SW Coastal Laizhou Bay, Bohai Sea, China: A Comprehensive Assessment Based on the Analysis of Heavy Metals
Source: PLoS One. 2015 Mar 27;10(3):e0122190. doi: 10.1371/journal.pone.0122190 (PMC4376849; doi:10.1371/journal.pone.0122190)
Supplement: S3 Table — (PDF) [file pone.0122190.s003.pdf]

S3 Table. Mean PEL quotient data.

Site	Mean PEL quotient	
	May-Jun.	Sept.-Oct.
YHH3	0.39	0.32
YHH2	0.55	
YHH1	0.56	0.22
GLH3	0.28	0.57
GLH2	0.32	0.28
GLH1	0.36	0.32
YHH-GLH	0.35	0.32
ZMH2	0.24	0.31
ZMH1	0.30	0.47
ZM-YHH	0.28	0.28
L1	0.20	0.23
L2	0.15	0.18
L3	0.20	0.21
L4	0.17	0.20
L5	0.21	0.22
MH6	0.18	0.17
MH5	0.39	0.33
XQH4	0.66	0.74
XQH3	0.63	0.86

Site	Mean PEL quotient	
	May-Jun.	Sept.-Oct.
XQH2	0.38	0.39
XQH1	0.34	0.33
K1	0.26	0.28
K2	0.21	0.25
K3	0.27	0.21
MH4	0.39	0.42
MH3	0.45	
MH2	0.20	0.18
MH1	0.55	0.31
J1	0.16	0.20
J2	0.19	0.23
J3	0.22	0.25
J4	0.27	0.28
BLH3	0.22	0.16
BLH2	0.23	0.25
BLH1	0.17	0.27
I1	0.21	0.23
I2	0.17	0.22

Site	Mean PEL quotient	
	May-Jun.	Sept.-Oct.
I3	0.21	0.21
DH2	0.38	8.46
DH1	0.36	1.14
YH5	0.22	0.24
YH4	0.33	0.45
YH3	0.24	0.23
YH2	0.23	0.24
YH1	0.19	0.19
H1	0.24	0.26
H2	0.21	0.23
H3	0.22	0.26
WH3	0.29	0.21
WH2	0.32	0.23
WH1	0.24	0.23
JLH2	0.33	0.28
JLH1	0.14	0.15
