# Supplementary material for: Sediment Quality of the SW Coastal Laizhou Bay, Bohai Sea, China: A Comprehensive Assessment Based on the Analysis of Heavy Metals
Source: PLoS One. 2015 Mar 27;10(3):e0122190. doi: 10.1371/journal.pone.0122190 (PMC4376849; doi:10.1371/journal.pone.0122190)
Supplement: S4 Table — (PDF) [file pone.0122190.s004.pdf]

S4 Table. I_{geo} data.

Site	Cd		Cr		Cu		Ni		Pb		Zn	
	May-Jun.	Sept.-Oct.	May-Jun.	Sept.-Oct.	May-Jun.	Sept.-Oct.	May-Jun.	Sept.-Oct.	May-Jun.	Sept.-Oct.	May-Jun.	Sept.-Oct.
YHH3	1.34	0.90	0.07	0.30	0.97	-0.58	-0.38	0.16	-0.12	-0.91	0.11	-0.54
YHH2	2.34		0.28		1.78		-0.10		0.48		0.41	
YHH1	1.37	0.91	0.38	0.01	1.36	-1.24	0.28	-0.48	1.02	-1.30	0.44	-1.23
GLH3	1.08	2.02	0.23	1.16	-1.00	0.18	-0.05	1.05	-0.89	0.02	-0.81	0.23
GLH2	1.33	0.89	0.37	0.25	-0.84	-0.86	0.14	-0.06	-0.75	-0.99	-0.56	-0.77
GLH1	1.31	0.80	0.34	0.17	-0.22	-0.41	0.29	0.14	-0.59	-0.85	-0.37	-0.45
YHH-GLH	1.41	0.67	0.23	0.36	-0.99	-0.55	-0.14	0.19	-0.90	-0.88	0.81	-0.50
ZMH2	1.18	0.68	-0.07	0.11	-0.93	0.48	-0.50	-0.40	-0.75	-0.76	-0.91	-0.55
ZMH1	1.50	1.12	0.30	-0.02	-0.94	-1.18	-0.05	-0.36	-0.95	-1.23	-0.28	2.01
ZM-YHH	1.05	1.27	0.30	0.34	-0.98	-1.19	-0.10	-0.20	-0.71	-0.50	-0.80	-0.83
L1	0.73	0.91	-0.08	0.01	-2.01	-1.96	-0.70	-0.47	-0.95	-0.61	-1.42	-0.92
L2	0.22	1.62	-0.51	-0.40	-2.28	-2.87	-1.19	-0.83	-1.40	-1.18	-1.67	-1.36
L3	0.77	0.96	0.04	0.19	-1.99	-2.57	-0.64	-0.54	-1.11	-1.68	-1.49	-0.96
L4	0.42	0.58	-0.21	0.02	-2.12	-1.69	-0.93	-0.90	-1.24	-0.94	-1.62	-1.18
L5	0.67	0.87	0.08	0.12	-1.66	-1.62	-0.59	-0.52	-1.07	-0.83	-1.31	-1.51
MH6	1.85	0.30	-0.14	-0.46	-2.03	-2.16	-1.01	-0.60	-1.18	-1.44	-1.73	-1.71
MH5	1.41	1.52	0.78	0.78	-0.56	-0.74	-0.10	-0.27	-0.50	-0.72	0.57	-0.18
XQH4	2.12	2.10	1.70	1.60	0.29	0.71	0.04	0.24	0.14	0.11	1.74	2.05
XQH3	2.09	2.33	1.77	1.87	0.20	0.96	0.25	0.56	-0.08	0.71	1.40	2.07
XQH2	1.58	1.42	0.65	0.77	-0.42	-0.26	0.32	-0.03	-0.63	-0.65	-0.06	0.43
XQH1	1.58	1.42	0.71	0.49	-0.71	-0.56	-0.17	-0.08	-0.69	-0.81	-0.06	-0.10
K1	0.71	0.90	0.37	0.45	-1.64	-1.33	-0.66	-0.49	-1.12	-0.83	-0.07	0.00
K2	0.70	0.90	0.25	0.37	-1.77	-1.77	-0.72	-0.43	-0.95	-0.78	-1.26	-0.91

Site	Cd		Cr		Cu		Ni		Pb		Zn	
	May-Jun.	Sept.-Oct.	May-Jun.	Sept.-Oct.	May-Jun.	Sept.-Oct.	May-Jun.	Sept.-Oct.	May-Jun.	Sept.-Oct.	May-Jun.	Sept.-Oct.
K3	0.75	1.61	0.06	-0.06	-0.87	-1.68	-0.17	-0.66	-0.59	-0.99	-0.83	-1.37
MH4	1.52	1.45	0.82	1.07	-0.79	-0.79	0.51	0.56	-0.44	-0.65	-0.51	-0.34
MH3	1.88		0.91		-0.30		0.64		-0.17		-0.31	
MH2	0.54	0.90	-0.20	-0.18	-1.45	-1.97	-0.64	-0.87	-1.13	-1.28	-1.43	-1.45
MH1	2.71	0.63	0.32	0.05	1.36	-0.01	-0.20	-0.48	-0.73	-0.50	1.32	0.07
J1	-0.13	1.69	-0.44	-0.30	-2.30	-1.82	-0.99	-1.11	-1.16	-0.70	-1.39	-0.96
J2	0.29	1.41	-0.15	0.03	-1.86	-1.46	-0.74	-0.44	-0.97	-0.80	-1.36	-1.33
J3	0.61	0.87	0.01	0.19	-1.51	-1.54	-0.50	-0.23	-0.86	-1.04	-1.17	-1.01
J4	0.91	1.09	0.13	0.06	-0.95	-0.86	-0.15	-0.06	-0.61	-0.57	-0.80	-0.73
BLH3	1.28	0.11	0.02	-0.37	-1.37	-2.09	-0.65	-0.99	-0.91	-1.27	-1.09	-1.69
BLH2	0.86	1.11	-0.13	-0.08	-0.17	-0.63	-0.75	-0.21	-1.02	-1.09	-1.48	-1.18
BLH1	1.16	-0.11	-0.34	-0.33	-2.06	-1.97	-1.09	-0.78	-1.35	-1.20	-1.31	0.80
I1	0.81	1.07	0.03	-0.06	-1.72	-2.04	-0.66	-0.39	-0.92	-0.67	-1.12	-0.94
I2	0.33	0.50	-0.44	-0.28	-2.01	-1.77	-0.89	-0.64	-1.06	-1.03	-1.44	-0.40
I3	0.44	0.68	-0.14	-0.03	-1.52	-1.26	-0.59	-0.67	-0.91	-1.15	-1.02	-1.05
DH2	1.19	1.23	0.22	0.21	-0.05	0.37	-0.05	0.43	-0.63	-0.80	0.65	6.96
DH1	1.31	2.34	-0.09	0.27	-0.55	0.33	-0.73	-0.53	-0.89	-0.13	1.25	3.70
YH5	1.32	1.41	-0.25	-0.03	-1.75	-1.42	-0.66	-0.57	-0.90	-0.68	-0.65	-0.80
YH4	1.40	1.29	-0.09	0.65	-1.68	-1.64	-0.75	0.90	-0.78	-1.23	1.18	0.59
YH3	0.88	2.06	-0.58	0.31	-0.90	-2.21	-0.98	-1.08	-1.04	-1.20	0.26	-0.27
YH2	0.89	0.88	0.08	0.01	-1.45	-1.53	-0.40	-0.52	-0.88	-1.04	-1.13	-0.37
YH1	1.90	1.40	0.18	0.07	-2.12	-2.36	-1.08	-0.85	-1.25	-1.20	-1.48	-1.47
H1	0.10	0.30	-0.54	-0.40	-2.00	-1.85	-0.89	-0.70	1.04	1.06	-1.19	-1.13

Site	Cd		Cr		Cu		Ni		Pb		Zn	
	May-Jun.	Sept.-Oct.	May-Jun.	Sept.-Oct.	May-Jun.	Sept.-Oct.	May-Jun.	Sept.-Oct.	May-Jun.	Sept.-Oct.	May-Jun.	Sept.-Oct.
H2	0.76	0.97	0.09	0.20	-1.83	-1.81	-0.74	-0.53	-0.89	-0.80	-1.18	-0.93
H3	0.93	0.92	0.11	0.06	-1.58	-1.20	-0.61	-0.56	-0.85	-1.18	-1.12	0.01
WH3	1.30	1.66	0.33	0.29	-1.05	-2.00	-0.06	-0.85	-0.48	-0.95	-1.13	-1.47
WH2	1.56	1.34	0.36	0.19	-0.49	-1.40	0.02	-0.44	-0.58	-0.98	-0.79	-1.45
WH1	1.09	1.39	0.10	0.14	-0.96	-1.45	-0.49	-0.50	-0.92	-1.00	-1.26	-1.47
JLH2	1.04	1.67	0.03	-0.21	-1.22	-1.40	0.54	-0.58	-0.73	-0.74	-0.86	0.40
JLH1	0.74	0.26	-0.76	-0.70	-2.19	-1.77	-1.37	-1.10	-1.20	-1.25	-1.87	-1.82
