# Supplementary material for: Sediment Quality of the SW Coastal Laizhou Bay, Bohai Sea, China: A Comprehensive Assessment Based on the Analysis of Heavy Metals
Source: PLoS One. 2015 Mar 27;10(3):e0122190. doi: 10.1371/journal.pone.0122190 (PMC4376849; doi:10.1371/journal.pone.0122190)
Supplement: S5 Table — (PDF) [file pone.0122190.s005.pdf]

S5 Table. EF data.

Site	Cd		Cr		Cu		Ni		Pb		Zn	
	May-Jun.	Sept.-Oct.	May-Jun.	Sept.-Oct.	May-Jun.	Sept.-Oct.	May-Jun.	Sept.-Oct.	May-Jun.	Sept.-Oct.	May-Jun.	Sept.-Oct.
YHH3	6.09	3.54	2.51	2.33	4.71	1.27	1.84	2.11	2.22	1.01	2.59	1.30
YHH2	11.6		2.79		7.87		2.14		3.20		3.05	
YHH1	5.11	4.91	2.57	2.63	5.05	1.11	2.39	1.88	3.99	1.07	2.67	1.12
GLH3	4.04	4.23	2.24	2.32	0.95	1.18	1.85	2.15	1.03	1.05	1.10	1.22
GLH2	4.46	3.94	2.29	2.52	0.99	1.17	1.95	2.03	1.05	1.07	1.20	1.25
GLH1	4.37	3.23	2.23	2.09	1.52	1.40	2.17	2.04	1.17	1.03	1.36	1.36
YHH-GLH	5.57	2.93	2.45	2.36	1.06	1.26	1.90	2.10	1.12	1.00	3.66	1.30
ZMH2	4.52	3.41	1.90	2.30	1.04	2.98	1.41	1.62	1.19	1.26	1.06	1.46
ZMH1	5.70	5.25	2.48	2.37	1.05	1.06	1.94	1.88	1.04	1.03	1.65	9.70
ZM-YHH	3.64	4.58	2.16	2.41	0.89	0.83	1.63	1.66	1.07	1.34	1.01	1.07
L1	3.57	3.66	2.04	1.97	0.53	0.50	1.33	1.40	1.12	1.27	0.81	1.03
L2	2.25	5.78	1.35	1.42	0.40	0.26	0.85	1.06	0.73	0.83	0.61	0.73
L3	3.63	3.87	2.19	2.26	0.54	0.34	1.36	1.37	0.98	0.62	0.76	1.02
L4	2.95	3.37	1.91	2.28	0.51	0.70	1.17	1.21	0.94	1.17	0.72	0.99
L5	3.27	3.52	2.17	2.08	0.65	0.62	1.37	1.34	0.98	1.08	0.83	0.67
MH6	7.69	4.07	1.94	2.40	0.53	0.74	1.06	2.18	0.94	1.22	0.64	1.01
MH5	5.06	7.38	3.27	4.41	1.29	1.55	1.78	2.13	1.34	1.56	2.81	2.28
XQH4	8.81	8.20	6.60	5.81	2.48	3.12	2.10	2.26	2.24	2.06	6.81	7.95
XQH3	9.00	8.99	7.22	6.57	2.43	3.48	2.53	2.64	2.00	2.94	5.58	7.50
XQH2	6.48	6.15	3.41	3.91	1.63	1.92	2.71	2.25	1.40	1.46	2.08	3.09
XQH1	6.38	6.93	3.49	3.65	1.31	1.76	1.90	2.46	1.32	1.48	2.05	2.43
K1	3.49	4.67	2.75	3.41	0.69	0.99	1.35	1.78	0.98	1.41	2.03	2.50
K2	3.46	3.71	2.52	2.57	0.62	0.58	1.29	1.48	1.10	1.16	0.89	1.06

Site	Cd		Cr		Cu		Ni		Pb		Zn	
	May-Jun.	Sept.-Oct.	May-Jun.	Sept.-Oct.	May-Jun.	Sept.-Oct.	May-Jun.	Sept.-Oct.	May-Jun.	Sept.-Oct.	May-Jun.	Sept.-Oct.
K3	2.87	4.93	1.78	1.56	0.94	0.51	1.51	1.02	1.14	0.81	0.96	0.63
MH4	5.25	5.74	3.24	4.39	1.06	1.22	2.61	3.09	1.35	1.34	1.29	1.65
MH3	6.22		3.17		1.37		2.62		1.50		1.36	
MH2	2.91	5.62	1.74	2.66	0.73	0.77	1.28	1.65	0.91	1.24	0.75	1.10
MH1	18.0	4.14	3.42	2.76	7.04	2.65	2.39	1.91	1.66	1.89	6.86	2.80
J1	2.02	7.68	1.63	1.94	0.45	0.68	1.12	1.11	0.99	1.48	0.85	1.23
J2	2.58	5.82	1.92	2.24	0.58	0.80	1.27	1.62	1.08	1.26	0.83	0.88
J3	3.08	3.43	2.03	2.15	0.71	0.65	1.43	1.61	1.11	0.92	0.90	0.94
J4	3.31	3.89	1.93	1.91	0.92	1.01	1.59	1.76	1.16	1.23	1.01	1.11
BLH3	4.90	3.45	2.05	2.48	0.78	0.75	1.29	1.61	1.07	1.33	0.95	0.99
BLH2	4.62	6.80	2.32	2.99	2.26	2.03	1.51	2.73	1.25	1.48	0.91	1.40
BLH1	5.44	2.37	1.92	2.05	0.58	0.66	1.14	1.50	0.95	1.12	0.98	4.46
I1	3.62	4.57	2.11	2.09	0.63	0.53	1.30	1.66	1.09	1.37	0.95	1.13
I2	2.69	3.37	1.58	1.97	0.53	0.70	1.15	1.54	1.03	1.17	0.79	1.81
I3	2.87	3.37	1.92	2.07	0.74	0.88	1.41	1.32	1.13	0.95	1.04	1.02
DH2	4.04	8.97	2.07	4.41	1.71	4.93	1.71	5.16	1.15	2.20	2.78	475
DH1	7.10	13.4	2.69	3.18	1.95	3.32	1.73	1.83	1.55	2.41	6.83	34.4
YH5	5.95	5.42	2.01	1.99	0.71	0.76	1.51	1.37	1.28	1.27	1.52	1.17
YH4	5.44	6.09	1.95	3.93	0.65	0.80	1.23	4.67	1.21	1.07	4.68	3.75
YH3	4.00	10.5	1.46	3.13	1.16	0.55	1.11	1.20	1.06	1.10	2.61	2.11
YH2	3.45	3.67	1.97	2.01	0.69	0.69	1.41	1.40	1.02	0.97	0.86	1.55
YH1	8.28	6.27	2.52	2.49	0.51	0.46	1.05	1.31	0.94	1.03	0.80	0.86
H1	2.31	2.92	1.49	1.80	0.54	0.66	1.16	1.46	4.42	4.94	0.95	1.09

Site	Cd		Cr		Cu		Ni		Pb		Zn	
	May-Jun.	Sept.-Oct.	May-Jun.	Sept.-Oct.	May-Jun.	Sept.-Oct.	May-Jun.	Sept.-Oct.	May-Jun.	Sept.-Oct.	May-Jun.	Sept.-Oct.
H2	3.53	5.26	2.21	3.08	0.59	0.76	1.25	1.85	1.12	1.54	0.92	1.41
H3	3.92	5.30	2.21	2.91	0.69	1.22	1.35	1.90	1.14	1.23	0.95	2.82
WH3	5.46	8.08	2.79	3.13	1.07	0.64	2.13	1.42	1.59	1.32	1.01	0.92
WH2	6.12	8.59	2.67	3.87	1.47	1.29	2.09	2.51	1.38	1.73	1.20	1.25
WH1	5.59	7.28	2.82	3.05	1.35	1.01	1.87	1.96	1.39	1.38	1.09	1.01
JLH2	6.10	6.58	3.04	1.79	1.27	0.78	4.33	1.39	1.79	1.25	1.64	2.74
JLH1	4.68	3.08	1.65	1.59	0.62	0.75	1.09	1.20	1.22	1.09	0.77	0.73
